# Mechanical properties of DNA-like polymers

**DOI:** 10.1093/nar/gkt808

**Published:** 2013-09-05

**Authors:** Justin P. Peters, Shweta P. Yelgaonkar, Seergazhi G. Srivatsan, Yitzhak Tor, L. James Maher

**Affiliations:** ^1^Department of Biochemistry and Molecular Biology, Mayo Clinic College of Medicine, 200 First St. SW, Rochester, MN 55905, USA, ^2^Indian Institute of Science Education and Research, 900, NCL Innovation Park, Dr. Homi Bhabha Road, Pune 411008, India and ^3^Department of Chemistry and Biochemistry, University of California, San Diego, La Jolla, CA 92093, USA

## Abstract

The molecular structure of the DNA double helix has been known for 60 years, but we remain surprisingly ignorant of the balance of forces that determine its mechanical properties. The DNA double helix is among the stiffest of all biopolymers, but neither theory nor experiment has provided a coherent understanding of the relative roles of attractive base stacking forces and repulsive electrostatic forces creating this stiffness. To gain insight, we have created a family of double-helical DNA-like polymers where one of the four normal bases is replaced with various cationic, anionic or neutral analogs. We apply DNA ligase-catalyzed cyclization kinetics experiments to measure the bending and twisting flexibilities of these polymers under low salt conditions. Interestingly, we show that these modifications alter DNA bending stiffness by only 20%, but have much stronger (5-fold) effects on twist flexibility. We suggest that rather than modifying DNA stiffness through a mechanism easily interpretable as electrostatic, the more dominant effect of neutral and charged base modifications is their ability to drive transitions to helical conformations different from canonical B-form DNA.

## INTRODUCTION

Double-helical DNA has unique attributes including its high thermal stability, high negative charge density and strong resistance to both bending and twisting. The prevailing wormlike chain (WLC) model has been useful in describing DNA behavior over lengths between ∼200 base pairs (bp) and ∼2000 bp (1,2). The WLC model can be used to express the cyclization *J*-factor (intramolecular concentration of DNA termini properly aligned for enzymatic ligation), as a function of DNA contour length and three parameters describing the DNA double helix: persistence length (*P*), helical repeat (*γ*_0_) and torsional rigidity (*C*). The *P* is a measure of the resistance of the polymer to bending, the *γ*_0_ of DNA defines the number of base pairs per helical turn and *C* refers to the resistance of the helix to changes in helical twist.

The impact of sequence on DNA mechanical properties has been well studied (3). It has long been appreciated that A/T versus G/C sequence content can influence flexibility relative to generic sequences (4), with an A/T rich nucleosome positioning sequence displaying decreases in *P* (from 51 nm for generic B-DNA to 26.5 nm), *γ*_0_ (from 10.45 bases/turn for generic B-DNA to 10.3 bases/turn) and *C* (from 2.4 × 10^−^^19 ^erg-cm for generic B-DNA to 1.5 × 10^−^^19 ^erg-cm) (5). In other studies, *C* measured for DNAs with different base compositions shows only a slight sequence dependence (6), while *γ*_0_ (10.27–10.76 bases/turn) and *P* (40.9–56.0 nm) of DNA both depend strongly on A/T versus G/C content (7). Molecular dynamics simulations also suggest sequence dependence can cause a 10–20% change in local bending rigidity (8). However, the relationship between stacking characteristics and bending mechanics is not straightforward. The stacking characteristics of the 10 natural DNA dimeric steps have been well studied using x-ray crystallographic and computational studies: the ‘rigid’ dimeric steps purine–pyrimidine (RY) and purine–purine (RR:YY) are characterized by a significant overlap of their aromatic rings and strong stacking interactions, while the ‘flexible’ pyrimidine–purine (YR) steps display distinct geometry and weaker stacking (9). Analysis of these nearest-neighbor effects with respect to DNA mechanical properties (7) reveals that while the ‘flexible’ step TA is also mechanically flexible (*P* = 44.7 nm), the ‘flexible’ step CG is the most mechanically rigid (*P* = 56.0 nm). Similarly, the ‘rigid’ step AC is mechanically rigid (*P* = 55.4 nm), although the ‘rigid’ step AT is the most mechanically flexible (*P* = 40.9 nm). Clearly, local dimer step conformational flexibility in crystallography is not the same as global mechanical bending flexibility.

Moreover, if the sequence-dependent contribution to DNA *P* accounts for ∼15% of the full *P* (as suggested from the range above), then the other ∼85% of *P* is not explained by sequence-dependent differences in stacking. Is there a ‘baseline’ contribution of stacking that is present in every kind of base pair, or a dominant contribution of electrostatic stiffness, or combinations of both? The thermal stability of double-helical DNA involves pairing of complementary bases and stacking of adjacent base pairs (10), yet it remains unclear if these base interactions play the dominant role in DNA bend and twist stiffness, as repulsive forces between negatively charged backbone phosphodiester linkages have also been implicated in conferring DNA mechanical rigidity through electrostatic tension (11). Thus, *why* different DNA sequences have different mechanical properties is unknown.

Theoretical models for the origin of DNA stiffness show no consensus, with the role of electrostatic effects ranging from negligible to dominant. For example, by applying a uniform charge density to the WLC model for suitably long DNAs, Odijk-Skolnick-Fixman theory (12,13) separated the persistence length of DNA into two additive components, one electrostatic and one inherent (nonelectrostatic). In this theory, the electrostatic component plays a relatively minor role in overall DNA stiffness. Other attempts have been made to augment the existing WLC theory for cyclization with an electrostatic component based on counterion condensation (CC) (14). This theory predicts a striking electrostatic contribution to DNA stiffness at low salt, but a much smaller contribution under physiological conditions. Toward the middle of the spectrum, recent all-atom and course-grained molecular dynamics simulations predict a significant contribution of electrostatic forces to DNA stiffness, comparable with that of nonelectrostatic interactions (15). Other calculations suggest that electrostatic forces make a substantial contribution to the energetics opposing the bending of fully charged DNAs into small circles (16). Counterion distributions in the DNA grooves have also been suggested as important electrostatic modulators determining local DNA mechanics (17–19). In the extreme, Manning’s ‘line of charge’ model offers the quantitative prediction that electrostatic forces make the dominant contribution to DNA stiffness (11). Thus, the relative contributions of base stacking and electrostatic tension to DNA rigidity continue to be debated theoretically.

It is challenging to experimentally isolate the roles of nonelectrostatic stiffening forces (including base pair stacking) from electrostatic forces. Hagerman and coworkers investigated the role of stacking in the absence of one charged DNA backbone using a so-called ‘gapped meroduplex’ (based on an unstructured single-stranded polypyrimidine region within a double helix). This molecule was shown to be much more flexible than the equivalent full duplex, but the flexibility returned to normal on addition of complementary free purine bases, suggesting that base pair stacking is the dominant contributor to DNA stiffness (20). On the other hand, laterally asymmetric modification of DNA charge induces DNA bending in both experiments and simulations, supporting a role for electrostatic effects in DNA mechanics (21–25).

**Figure 1.**
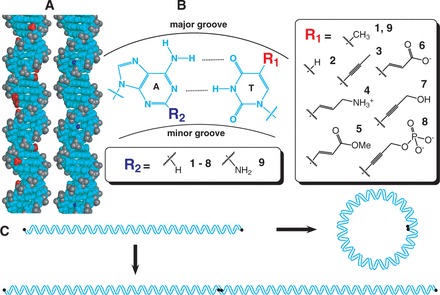
Experimental design. (**A**). Three-dimensional structure of B-form DNA (26). The central stacked DNA base pairs and deoxyribose sugars are uncharged (cyan), while each phosphodiester linkage carries a negative charge (gray). Seven of the eight tested base modifications replace the methyl group (red) of thymine bases in the DNA major groove, while one modification replaces the N_2_ proton (blue) of adenine bases in the DNA minor groove. (**B**). Chemical structure of an A·T base pair. Base modifications occur either at the 5 position of thymine (compare **1** with **2**-**8**) or the 2 position of adenine (**9**). Based on the p*K*_a_ values of the isolated functional groups, modifications **4**, **6**, and **8** alter DNA charge at neutral pH. (**C**). DNA ligase-catalyzed cyclization kinetics experiments to analyze DNA bend and twist stiffness. End-labeled (black circle) linear DNA fragments (∼200 bp, top) are detected when they either cyclize (right) or multimerize (bottom). The readout of these experiments is a ring closure probability (*J*-factor), which can be interpreted using the WLC model to estimate DNA mechanical parameters.

Here we present an unbiased experimental approach to explore the origins of the mechanical properties of DNA-like polymers. We synthesized eight DNA-like duplexes with chemical modifications that preserve base pairing while decorating the DNA grooves to alter charge, base stacking or both (Figure 1A). Neutral and charged DNA base modifications were designed in the DNA grooves at A·T base pairs (Figure 1B) in such a way that the modifications were scattered symmetrically over ∼200 bp in an intrinsically straight context created by 5-bp tandem repeats of nonrepetitive sequences such that any deviation in helix axis induced by sequence/analog effects in the first half of each helical turn is compensated by a corresponding correction in the second half of each helical turn (27). We then characterized the polymers and measured the bend and twist stiffness of each using ligase-catalyzed cyclization kinetics experiments (Figure 1C).

## MATERIALS AND METHODS

### dNTP analogs

Modified dUTP analogs **2**, **3** and **4** and the dATP analog **9** were purchased from TriLink BioTechnologies. Synthesis of the remaining dUTP analogs (**5**, **6**, **7** and **8**) is described in Supplementary Data S1.

### Polymerase chain reaction amplification of modified DNAs

pUC19-based plasmids containing intrinsically straight ∼200-bp sequences (27) were subjected to site-directed mutagenesis to obtain suitable restriction sites (Supplementary Data S2). Polymerase chain reaction (PCR) products (∼400 bp) containing these intrinsically straight sequences flanked by *Nar*I sites were amplified using primers LJM-3222 (5′-G
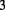
TA
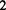
CGC
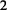
AG
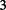
T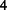) and LJM-3223 (5′-TGTGAGT
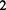
AGCTCACTCAT
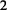
AG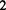) (Integrated DNA Technologies).

For analog **2**, PCR reactions (100 µl) included 20 ng plasmid template (or alternatively 20 ng of purified PCR product from a previous reaction), 0.4 mM forward and reverse primers, 100 mg/ml bovine serum albumin (BSA), *Taq* DNA polymerase buffer (Invitrogen), 2 mM MgCl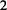, 0.2 mM each dNTP (with dTTP completely replaced by the analog triphosphate) and 5 U *Taq* DNA polymerase (Invitrogen). Cycle conditions were 98°C (3 min), 30 cycles of [94°C (30 s), 60°C (30 s) and 72°C (45 s)], followed by 72°C (5 min).

For analogs **3**, **4**, **5**, **6** and **9**, PCR reactions (100 µl) included 20 ng template, 0.4 mM forward and reverse primers, PrimeSTAR GC buffer (Takara), 0.2 mM each dNTP (again with dTTP completely replaced with analog, except for variant **9**, which completely replaced dATP), 2 M betaine (Sigma-Aldrich) and 5 U PrimeSTAR HS DNA polymerase (Takara). Cycle conditions were 98°C (3 min), 30 cycles of [98°C (15 s), 60°C (5 s) and 72°C (45 s)], followed by 72°C (5 min).

For analogs **7** and **8**, PCR reactions (100 µl) included 20 ng template, 0.4 mM forward and reverse primers, *Pwo* PCR buffer (Roche), GC-rich solution (Roche), 0.2 mM each dNTP (dTTP completely replaced with analog), 2 M betaine (Sigma-Aldrich) and 5 U *Pwo* SuperYield DNA Polymerase (Roche). Cycle conditions were 98°C (3 min), 30 cycles of [98°C (1 min), 60°C (2 min) and 72°C (8 min)], followed by 72°C (5 min). To generate DNA where only one strand contained analog **8**, the previous conditions were modified so that the template was replaced with the desired amount of unmodified PCR product and the number of cycles was reduced from 30 to a single extension cycle.

### Ion exchange chromatography

Fast-performance liquid chromatography was performed using the BioLogic DuoFlow QuadTec system (BioRad). The chromatography was monitored with a ultraviolet detector at 260 nm and a conductivity meter. Samples (∼1 µg in 25 µl) were loaded on an anion exchange MonoQ HR 5/5 column (0.5 × 5 cm; Amersham Biosciences) equilibrated with 20 mM Tris–HCl, pH 8 (buffer A), then eluted at a 1 ml/min flow rate with buffer B (buffer A plus 1 M NaCl) using the following elution conditions: 0–3 min, 50% B isocratic; 3–28 min, linear gradient 50–100% B; 28–30 min, 100% B isocratic, and, finally, reconditioning of the column with 50% B isocratic for 5 min. PCR conditions above were modified as follows: primers LJM-4449 (5′-AGC
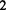
TAGC
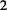
TATGACATGAC) and LJM-4450 (5′-GAG
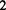
TGAG
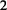
T
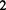
GCAT
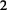
GCAT) and 98-bp DNA template pJ1923 (top strand 5′-AGC
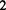
TAGC
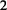
TATGACATGACACGT
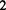
ACGAC
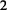
AGAC
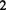
AGCTGCACTGCAGACTG
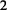
ACTGACGCTAGCTGACTGTACTGTATGCA
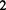
TGCA
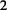
C
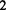
TCAC
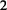
TC). Samples of length 98 bp were used so that elution occurred near the middle of the salt gradient (∼750 mM NaCl).

### Thermodynamic characterization of DNA-like polymers

Thermal denaturation experiments monitored SYBR Green I (Invitrogen) fluorescence using an ICycler thermocycler (BioRad) over a temperature range of 50–100°C, measurements collected every 0.1°C with a temperature slope of 24°C/h. Samples (20 µl) contained 100 ng DNA [∼30 nM 418-bp DNA (pJ1506) depending on extinction coefficient, Supplementary Data S2] in 10 mM sodium cacodylate, pH 6.6, with 10 mM NaCl and either 0.06×, 0.08×, 0.1× or 0.2× SYBR Green I (Invitrogen). The melting temperature (*T*_m_) and change in standard free energy (Δ*G*°), enthalpy (Δ*H*°) and entropy (Δ*S*°) of the dissociation reaction were determined from the thermal denaturation data using the van’t Hoff equation assuming a two-state transition model (28). Limitations and assumptions of the model are detailed in Supplementary Data S2. To eliminate the effects of potential binding affinity differences of the dye, each experiment was performed in triplicate for a given dye concentration, and dye-free values of the parameters were determined by linear extrapolation to zero dye concentration.

### Circular dichroism spectroscopy

Circular dichroism (CD) spectroscopy was performed using a J-810 spectropolarimeter (Jasco). Briefly, ultraviolet-CD spectra were acquired from 350–200 nm, taking measurements every 0.1 nm with a scanning speed of 5 nm/min. Sample temperature was maintained at 20°C throughout. Samples were monitored five times with the average of the five scans reported. Samples were analyzed in a 0.1-cm cuvette and prepared by diluting ∼25 µg of 417-bp DNA (pJ1741) into 300 µl of 10 mM phosphate buffer, pH 7.0, containing 1 M NaCl (final DNA concentration of ∼650 µM). Buffer contribution was subtracted from each sample.

### DNA cyclization kinetics assay

PCR products (from pJ1506 and pJ1741–pJ1750) were purified using QIAquick PCR Purification Kit (Qiagen) and then digested overnight with *Nar*I and phosphatase-treated with Antarctic Phosphatase under conditions recommended by the supplier (New England Biolabs, NEB). The reaction was heat inactivated for 20 min at 65°C followed by radioactive labeling for 1 h in T4 Polynucleotide Kinase (PNK) buffer (NEB) using 600 pmol of [γ-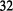
P]-ATP (PerkinElmer) and 40 U T4 PNK (NEB) and an additional heat inactivation for 20 min at 65°C. Samples (60 µl) were then loaded onto a 5% native polyacrylamide gel (29:1 acrylamide:bisacrylamide, BioRad) and visualized by exposure to BioMAX XR film (Kodak). The ∼200-bp restriction fragment was cut from the gel, crushed and eluted overnight at ∼22°C in 50 mM NaOAc, pH 7.0. The eluted DNA was precipitated from ethanol and quantified using a NanoDrop 1000 Spectrophotometer (Thermo Scientific).

Concentrations were determined at 260 nm using nearest neighbor molar extinction coefficients (29). Values for oligonucleotides containing modified residues were obtained in the following way: the individual extinction coefficients for all the nucleotides (Supplementary Data S2) were summed and compared with the sum from the corresponding natural sequence. The ratio of the two was used to scale the molar extinction coefficient of the natural sequence derived from nearest neighbor parameters. Because the content of modified residues is ∼25%, this estimation method is unlikely to generate large errors in concentration.

DNA ligase-catalyzed cyclization reactions were performed at ∼22°C with 1 nM DNA restriction fragment, T4 DNA ligation buffer (50 mM Tris–HCl, pH 7.5, 10 mM MgCl_2_, 1 mM ATP, 10 mM dithiothreitol; NEB) and a final concentration of 100 U/ml T4 DNA ligase (NEB). Aliquots (10 µl) were removed at 5, 10, 15 and 20 min time points, quenched by addition of EDTA to 20 mM and then analyzed by electrophoresis through 5% native polyacrylamide gels (29:1 acrylamide:bisacylamide, BioRad) in 0.5× TBE buffer (50 mM Tris base, 55 mM boric acid and 1 mM EDTA, pH 8.3), followed by drying and storage phosphor imaging.

Gel imaging was performed using a Typhoon FLA 7000 (GE Healthcare), and band quantitation was performed using R software (Version 2.14.2). After correction for background, signal intensities for individual species were normalized to the total signal for each gel lane. The resulting values were multiplied by the original DNA concentration to calculate the concentration of each species as a function of time. The subsequent method of *J*-factor determination involved a curve-fitting approach previously described (30) (see also Supplementary Data S3).

### WLC analysis

#### WLC model

The *J*-factor is reasonably approximated by the product of the bending (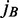) and twisting (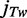) components (7). In this way, the ligase-mediated *J*-factor, expressed as a function of DNA contour length *L*, is defined explicitly by three parameters of the DNA: *P*, *γ*_0_ and *C*:
(1)
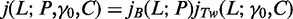



A semiempirical model for the *J*-factor uses the bending component of the *J*-factor found analytically (31)
(2)
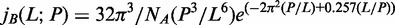



along with empirically determined expressions from Monte Carlo simulations (32,33)
(3)
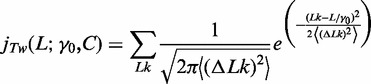

where 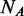 is Avogadro’s constant and the variance of the linking number (*Lk*) distribution for circular forms, 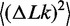, and is given by
(4)
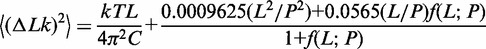

with
(5)
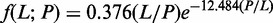

where *k* is the Boltzmann constant and *T* the absolute temperature. For ∼200-bp DNA molecules, only a limited set of topoisiomers (values of linking number *Lk*) make substantial contribution to 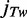. Thus, in practice, the sum is taken over the seven integers closest to 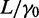.

#### Optimization

A weighted nonlinear least squares method was used to minimize the cost function *C*(**p**) with respect to the free parameters 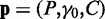

(6)
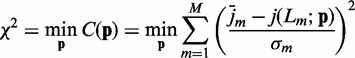

where *M* is the number of data points (i.e. the DNA lengths) being fit, and (corresponding to the *m*-th data point) 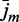 are experimentally determined mean *J*-factors, 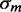 are standard deviations of 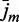 and 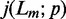 are theoretical predictions of the model. Optimizations were performed using a simplex and inductive search hybrid algorithm (34).

#### Monte Carlo estimation of uncertainty

Simulated data sets were generated from the experimental data by adding Gaussian noise, i.e. randomly sampling from the normal distributions 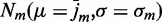. These simulated data sets (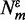 for 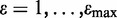) were then minimized against the WLC model to determine 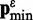.
(7)
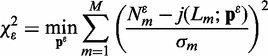



For a given parameter 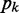, the standard deviation is estimated as
(8)
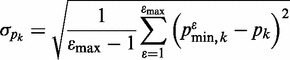

where 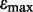 was set at 10 000 and 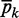 is the mean value
(9)
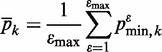



Additionally, the relative bias in the parameter 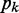 is given by
(10)
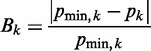



For each parameter, the relative bias was <2% (and typically much smaller), giving confidence in the method.

## RESULTS

### Preparation of DNA-like polymers

**Figure 2.**
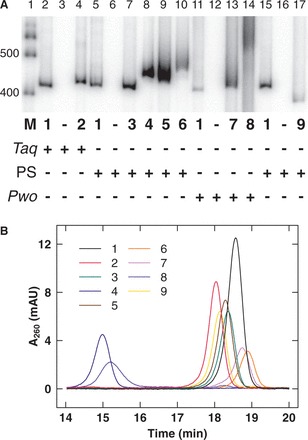
Characterization of DNA analogs. (**A**) PCR assays analyzed by 5% native polyacrylamide gel electrophoresis. Total PCR volume 100 µl: 20 ng 418-bp DNA template (pJ1506), 0.4 mM each LJM-3222 (5'-G
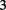
TA
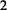
CGC
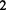
AG
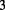
T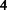) and LJM-3223 (5'-TGTGAGT
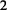
AGCTCACTCAT
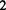
AG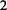), 0.2 mM each dNTP with indicated analog triphosphate (**1–9**) completely replacing appropriate dNTP, and 5 U DNA polymerase (indicated with plus symbol) with associated buffer and cycle conditions. *Taq* DNA polymerase (*Taq*) conditions: *Taq* DNA polymerase buffer with 100 mg/ml BSA and 2 mM MgCl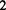; 98°C (3 min), 30 cycles of [94°C (30 s), 60°C (30 s), and 72°C (45 s)], 72°C (5 min). PrimeSTAR HS DNA polymerase (PS) conditions: PrimeSTAR GC buffer with 2 M betaine; 98°C (3 min), 30 cycles of [98°C (15 s), 60°C (5 s), and 72°C (45 s)], 72°C (5 min). *Pwo* SuperYield DNA Polymerase (*Pwo*) conditions: *Pwo* PCR buffer with GC-rich solution and 2 M betaine; 98°C (3 min), 30 cycles of [98°C (1 min), 60°C (2 min), and 72°C (8 min)], 72°C (5 min). Lane 1 is marker (M) DNA (100 bp DNA ladder, Invitrogen) with 400 - and 500-bp bands indicated. (**B**) Anion exchange chromatography of 98-bp DNA-like polymers (pJ1923). Following equilibration in 20 mM Tris–HCl, pH 8 (buffer A), samples were eluted over 25 min at a 1 ml/min flow rate in a linear gradient from 50 to 100% buffer B (buffer A plus 1 M NaCl). Eluent absorbance at 260 nm (milli-absorbance units) was monitored with elution time (min).

To create a collection of DNA-like polymers, we screened thermostable archaeal DNA polymerases for performance in PCR using modified deoxynucleotide triphosphates (35,36) and then optimized synthesis using a fractional factorial design to determine necessary PCR additives and conditions. Two polymerases (PrimeSTAR HS and *Pwo* SuperYield) proved most robust (Figure 2A). High fidelity amplification was achieved, with no product observed when a deoxynucleotide triphosphate analog was withheld (Figure 2A, lanes 3, 6, 12 and 16), and the ability to alternate between restriction endonuclease resistance and susceptibility depending on the presence or absence, respectively, of modification (Supplementary Data S2). Because DNA **8** migrated as a diffuse band (Figure 2A, lane 14), it was inferred that modification of all thymine residues was structurally disruptive. DNA **8** was therefore studied with only one modified strand in cyclization kinetics experiments. We showed analytically that the intended chemical modifications were present in the resulting polymers (Supplementary Data S2).

### Electrophoretic and ion exchange characterization of DNA-like polymers

Immediately evident from Figure 2A is the surprising fact that native polyacrylamide gel electrophoretic mobility is not simply predicted by the bare charges of the DNA-like polymers (35). For example, although analogs **6** and **8** increase the total negative charge while analog **4** decreases it, all retard electrophoretic mobility. Surprisingly, the only DNA-like polymer with increased electrophoretic mobility (**9**) does not alter DNA charge. This result calls into question current theories arguing that linear charge density governs DNA electrophoretic mobility. We emphasize that the dependence of electrophoretic mobility on charge may be complicated by other factors, including the mass, hydrophobicity, hydration and frictional properties of the appended functional group as well as the ionic composition and strength of the buffer. Gel electrophoresis of DNA-like polymers appears sensitive to the type of sieving medium and its potential for interaction with the functional groups.

It remains possible that this result reflects a fundamental prediction of CC theory (37): the extent of compensating CC is dictated by polymer charge density such that *polymers of varying charge density attain the same net charge after CC*. Perhaps the electrophoretic mobilities of DNA-like polymers largely reflect a constant *net* charge and nonelectrostatic features of the DNA-like polymers further fine-tune electrophoretic mobility.

We further characterized the electrostatic behavior of DNA-like polymers using anion exchange chromatography (Figure 2B). As observed for gel electrophoresis, the affinities of the DNA-like polymers for the positively charged ion exchange matrix did not correspond to their bare charge densities. Analogs **4** and **8** (with opposite effects on charge density) both display a decrease in elution time from the anion exchange column. This result perhaps again suggests that the polymers all attain the same net charge in solution owing to compensating CC. However, it is striking that the charged variants display the most extreme behaviors, as was the trend for gel electrophoresis. Taken together, these results challenge the simplicity of bare charge theories and highlight our incomplete understanding of these aspects of DNA electrostatics.

### Thermodynamic characterization of stacking and stability

The stacking propensity of various unpaired natural and analog nucleotides (X) was evaluated from thermodynamic measurements of the self-complementary DNA oligonucleotide 5′-XCGCGCG (38). All of the tested analogs showed favorable stacking of the dangling base (X), stabilizing the core hexamer duplex and increasing both *T*_m_ and annealing free energy (ΔG°) at 37°C (Supplementary Data S2). The measured stacking energies (ΔΔG°_37_) of all of the tested analogs equaled or exceeded the respective natural nucleotides (Supplementary Data S2).

Thermal denaturation studies of 418-bp duplexes showed that most of the DNA-like polymers displayed enhanced thermal stability (Table 1). Only the negatively charged DNA analogs **6** and **8** displayed both a decrease in *T*_m_ and less favorable annealing free energy change at 37°C (Supplementary Data S2). Overall, these data suggest that the DNA-like polymers have stabilities comparable with natural DNA.

**Table 1.** Parameters determined from analysis

DNA	*T*_m_ (°C)	*P* (nm)	*P*_Manning_ (nm)	*γ*_0_ (base/turn)	*C* (× 10^−19 ^erg-cm)	*P*_t_ (nm)
1	87.1 ± 0.5	51.5 ± 0.2	51.5	10.52 ± 0.01	2.27 ± 0.11	55.7 ± 2.6
2	85.3 ± 0.6	45.4 ± 0.4	–	10.47 ± 0.01	2.30 ± 0.13	56.5 ± 3.1
3	93.3 ± 0.7	42.9 ± 0.3	–	10.62 ± 0.01	3.07 ± 0.19	75.4 ± 4.7
4	91.5 ± 0.7	44.3 ± 0.1	38.1	10.62 ± 0.01	1.48 ± 0.05	36.4 ± 1.2
5	88.3 ± 0.3	41.8 ± 0.1	–	10.69 ± 0.01	0.85 ± 0.04	20.9 ± 1.0
6	86.5 ± 0.7	41.8 ± 0.1	64.6	10.51 ± 0.02	0.61 ± 0.03	14.9 ± 0.8
7	93.4 ± 0.3	50.3 ± 0.1	–	10.64 ± 0.01	1.09 ± 0.07	26.8 ± 1.6
8	86.8 ± 0.4	58.5 ± 0.2	58.1	10.57 ± 0.01	1.08 ± 0.08	26.5 ± 1.9
9	93.0 ± 0.5	49.5 ± 0.2	–	10.58 ± 0.01	3.24 ± 0.07	79.6 ± 1.8

The reported values of *T*_m_ from thermal denaturation of modified DNAs are the mean and standard deviation from three independent repeats. *P*, *γ*_0_ and *C* along with the related *P*_t_ are presented as mean ± standard deviation from Monte Carlo simulations. Manning’s theoretical charge-stiffness prediction for persistence length (*P*_Manning_) is shown for comparison (11).

### Structural characterization of DNA-like polymers

**Figure 3.**
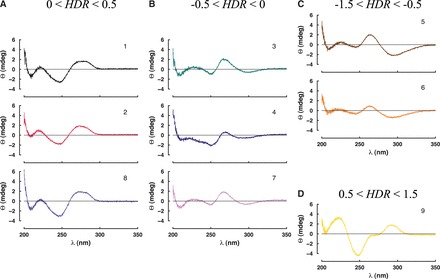
CD spectroscopy. CD spectra of 417-bp DNA-like polymers (pJ1741) in 10 mM phosphate buffer, pH 7.0, containing 1 M NaCl showing ellipticity (Θ) as a function of wavelength (λ) were divided into four groups based on *HDR* [*HDR* = Θ(λ_290_)/Θ(λ_201_)] in the ranges 0 < *HDR* < 0.5 (**A**), −0.5 < *HDR* < 0 (**B**), −1.5 < *HDR* < −0.5 (**C**) and 0.5 < *HDR* < 1.5 (**D**).

It is important to note that even base modifications that conserve normal DNA charge and base pairing might influence DNA structure and mechanical properties by steric, stacking and hydration effects. Therefore, we investigated the structures of the DNA-like polymers using CD spectroscopy (Figure 3). Interestingly, this analysis suggested that analog incorporation drove transitions to various helical conformations with differences from canonical B-form DNA. Four families of variant helical conformations are suggested from the CD analysis (Supplementary Data S2). Although considerable variation exists between the groups, all of the variants retain many B-type helical features. The first group includes natural DNA **1** as well as variants **2** and **8**. This group is the most similar to canonical B-DNA. The second group includes variants **3**, **4** and **7** whose negative CD peaks are shifted to longer wavelengths and positive peaks are shifted to shorter wavelengths with broadened crossovers. This second group exhibits unbalanced intensity for the positive versus negative CD peaks and displays a low intensity negative peak near 295 nm. For the third group (variants **5** and **6**), a high-intensity negative CD peak around 295 nm is pronounced. This feature is reminiscent of a Z-DNA signature. However, this group also displays positive CD peaks near 264 nm crossing over to negative peaks near 245 nm. These features, though shifted to shorter wavelengths, reflect a B-type CD signature. This partial inversion of CD spectra (here called Z-type character) has been previously noted for certain highly modified DNA-like polymers with three or four natural bases nearly completely replaced by analogs (36). Finally, adenine substitution by diaminopurine (variant **9**) has the most distinct CD spectrum. This variant (the only member of the fourth group) exhibits a positive CD peak shift to 292 nm and a deep negative peak at 248 nm with a shoulder and shallow crossover near 275 nm, suggesting A-type helical character. Partial A-type character has been previously reported for this substitution (39).

We note that ellipticity at 290 nm is loosely diagnostic of helical family. CD signal at this wavelength is known to be negative for Z-DNA, positive for A-DNA, and near zero (or slightly positive) for B-DNA (40,41). We therefore defined a helical diagnostic ratio, *HDR* (the ratio of CD ellipticities at wavelengths 290 and 201 nm), which can differentiate between the four groups above (Figure 3). *HDR* <0.5 indicates Z-type features, >0.5 indicates A-type features, between 0 and 0.5 indicates B-form DNA and between −0.5 and 0 indicates features between the previous Z-type and B-type. We emphasize that observed *HDR* values suggest variation within the B-form helix family, not conversion to A- or Z-DNA. Also, it is possible that conformational changes affect only subsequences within the test sequence.

### Mechanical characterization of DNA-like polymers

**Figure 4.**
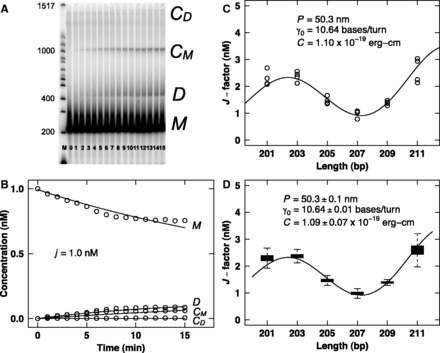
Example measurement of mechanical properties. (**A**) Cyclization time course for 207-bp DNA-like polymer **5** (pJ1744). DNA ligase-catalyzed cyclization reaction was performed at ∼22°C with 1 nM DNA restriction fragment, T4 DNA ligation buffer (50 mM Tris–HCl, pH 7.5, 10 mM MgCl_2_, 1 mM ATP, 10 mM dithiothreitol) and a final concentration of 100 U/ml T4 DNA ligase. Aliquots (10 µl) were removed at 1–15 min time points, quenched by addition of EDTA to 20 mM and then analyzed by electrophoresis through 5% native polyacrylamide gels in 0.5× TBE buffer (50 mM Tris base, 55 mM boric acid and 1 mM EDTA, pH 8.3). Gel lanes contains Invitrogen 100 bp DNA ladder (M), linear monomer without ligase (0) and increasing 1-min time points of the ligation reaction (1–15) showing the evolution of linear monomer (*M*), linear dimer (*D*), circular monomer (*C*_M_) and circular dimer (*C*_D_). Nearest molecular weight bands are indicated. (**B**) Cyclization kinetics analysis for 207-bp DNA-like polymer **5** (pJ1744). Fitting of data in (A) determines the *J*-factor, as previously described (30) (see also Supplementary Data S3). (**C**) WLC analysis for DNA-like polymer **5**. Fit of experimental *J*-factor data using the WLC model. (**D**). Monte Carlo estimation of uncertainty. Fit of simulated *J*-factor data based on (C) using the WLC model.

**Figure 5.**
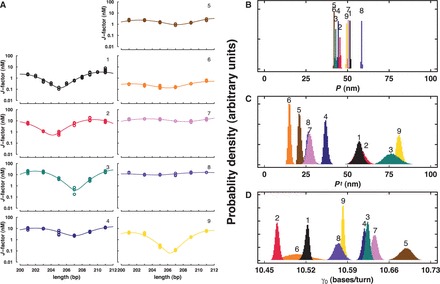
Summary of WLC analysis. *J*-factor curves for natural and DNA-like polymers (**A**) and parameter distributions for *P* (**B**), *P*_t_ (related to by *C* = *kTP*_t_) (**C**) and *γ*_0_ (**D**).

We then measured the mechanical properties of the DNA-like polymers using ligase-catalyzed cyclization experiments (Figure 1C and Supplementary Data S3). The stiffness of short ∼200-bp DNA molecules (*M*) is measured from the relative rates of intramolecular DNA cyclization to form monomeric circles (*C*_M_) versus intermolecular dimerization to form linear dimers (*D*). This ratio (*k*_C1_/*k*_D_) provides the cyclization *J*-factor, equivalent to the intramolecular concentration of one DNA terminus with respect to the other (Table 2) (1). Determination of these rates requires a cyclization timecourse (Figure 4A) and fitting data with a kinetic model (Figure 4B). Interestingly, ligation rate constants for cyclization and dimerization were observed to differ strikingly for different DNA analogs (Table 2), suggesting the possibility that ligation rate involves a one-dimensional ligase scanning step where the scanning rate is analog dependent (42). The *J*-factor data (Figure 5A) were then fit with the WLC model to estimate the mechanical parameters of the modified DNA-like polymers (Figure 4C). Assumptions and limitations of the WLC model were recently reviewed (43). From the spread of the experimental data, estimation of uncertainty was achieved using Monte Carlo simulations (Figure 4D). The results of this analysis are presented in Table 1 and Figure 5.

**Table 2.** Cyclization *J*-factor determined from kinetic rates *k*_C1_ and *k*_D_

DNA length (bp)	*J*-factor (nM)	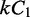 (× 10^−4^s^−1^)	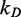 (× 10^−4 ^nM^−1^s^−1^)
DNA 1			
201	2.2 ± 0.2	13.9 ± 5.6	6.5 ± 3.1
202	1.5 ± 0.3	12.9 ± 4.8	9.2 ± 4.4
203	0.5 ± 0.1	5.3 ± 1.8	10.9 ± 4.5
204	0.3 ± 0.1	3.5 ± 1.5	10.9 ± 5.0
205	0.1 ± 0.1	2.1 ± 1.2	15.3 ± 7.0
206	0.3 ± 0.1	8.8 ± 1.3	26.6 ± 5.1
207	0.6 ± 0.1	5.7 ± 0.7	9.6 ± 1.5
208	1.2 ± 0.2	11.5 ± 2.0	9.7 ± 3.3
209	2.6 ± 0.7	24.2 ± 5.0	9.7 ± 3.1
210	3.4 ± 0.2	50.9 ± 9.8	14.9 ± 2.4
211	5.0 ± 1.5	48.3 ± 9.1	10.4 ± 3.5
DNA 2			
201	6.0 ± 0.9	13.3 ± 1.8	2.3 ± 0.4
203	1.2 ± 0.1	2.4 ± 0.2	2.1 ± 0.2
205	0.8 ± 0.3	1.9 ± 0.8	2.4 ± 0.7
207	5.6 ± 1.3	12.2 ± 4.5	2.1 ± 0.5
209	14.7 ± 4.6	24.7 ± 8.7	1.7 ± 0.2
211	9.1 ± 1.5	20.7 ± 6.0	2.3 ± 0.7
DNA 3			
201	17.6 ± 4.1	22.2 ± 8.3	1.3 ± 0.3
203	17.1 ± 2.4	25.4 ± 9.2	1.5 ± 0.4
205	4.2 ± 0.7	9.6 ± 5.8	2.4 ± 1.7
207	0.4 ± 0.3	0.9 ± 0.4	2.1 ± 0.3
209	3.4 ± 0.9	5.6 ± 2.9	1.7 ± 1.1
211	18.3 ± 3.8	31.0 ± 7.2	1.7 ± 0.1
DNA 4			
201	10.1 ± 2.1	21.1 ± 4.0	2.2 ± 0.8
203	9.7 ± 0.5	16.6 ± 4.2	1.7 ± 0.5
205	4.2 ± 1.8	11.0 ± 4.0	2.7 ± 0.4
207	2.3 ± 0.2	7.0 ± 3.2	3.0 ± 1.0
209	5.1 ± 0.6	12.4 ± 5.3	2.4 ± 0.8
211	12.3 ± 0.9	23.2 ± 4.1	1.9 ± 0.3
DNA 5			
201	2.3 ± 0.3	403 ± 31	177 ± 15
203	2.4 ± 0.2	443 ± 54	188 ± 24
205	1.5 ± 0.1	238 ± 23	163 ± 17
207	1.0 ± 0.1	171 ± 36	173 ± 17
209	1.4 ± 0.1	229 ± 36	165 ± 16
211	2.6 ± 0.5	486 ± 64	190 ± 26
DNA 6			
201	0.29 ± 0.02	119 ± 12	402 ± 24
203	0.24 ± 0.03	92 ± 25	384 ± 66
205	0.16 ± 0.04	64 ± 18	397 ± 53
207	0.16 ± 0.01	64 ± 15	402 ± 71
209	0.32 ± 0.01	103 ± 08	325 ± 29
211	0.57 ± 0.06	157 ± 15	274 ± 18
DNA 7			
201	13.5 ± 1.4	272 ± 05	20.4 ± 2.2
203	14.8 ± 0.4	294 ± 19	19.8 ± 1.9
205	13.4 ± 1.8	239 ± 10	18.0 ± 2.4
207	9.4 ± 0.1	200 ± 19	21.2 ± 1.7
209	8.5 ± 1.7	144 ± 22	17.6 ± 5.5
211	14.1 ± 2.1	262 ± 06	18.9 ± 2.9
DNA 8			
201	15.0 ± 1.6	288 ± 31	19.1 ± 0.1
203	9.8 ± 0.8	183 ± 32	19.0 ± 4.8
205	9.8 ± 0.8	108 ± 13	11.2 ± 2.1
207	10.4 ± 2.5	174 ± 26	17.8 ± 6.6
209	14.8 ± 1.6	233 ± 17	15.8 ± 1.9
211	15.8 ± 0.3	265 ± 29	16.8 ± 2.2
DNA 9			
201	4.2 ± 0.4	7.0 ± 0.8	1.7 ± 0.2
203	3.4 ± 0.7	2.4 ± 0.7	0.8 ± 0.4
205	0.3 ± 0.1	0.9 ± 0.2	3.3 ± 0.3
207	0.1 ± 0.1	0.3 ± 0.1	2.8 ± 0.3
209	1.7 ± 0.1	4.3 ± 0.4	2.5 ± 0.2
211	6.0 ± 0.5	7.9 ± 3.2	1.3 ± 0.6

Values are presented as mean ± standard deviation.

The measured bending *P* of natural DNA (**1**), 51.5 nm, is consistent with previous reports (27). The distribution of this parameter for each of the DNA-like polymers is shown in Figure 5B. Base modifications had remarkably subtle effects on bending stiffness, tending to soften the resulting polymers, with observed changes in *P* of <20%. Interestingly, there is no obvious correlation between effects on *P* and the bare charge of the DNA-like polymer.

The measured twist persistence length (*P*_t_) is shown in Figure 5C. This parameter is related to the *C*, by *C* = *kTP*_t_ where *k* is the Boltzmann constant and *T* the absolute temperature. Figure 5 and Table 1 show that, surprisingly, it is *P*_t_ that is most sensitive to chemical modification of the DNA bases. At its most extreme, *P*_t_ decreases >3-fold and exhibits an overall 5-fold range among the tested DNA-like polymers. Again, there is no correlation between the degree of twist modulation and the polymer charge. Interestingly, it has previously been reported that *P*_t_ is more sensitive to ethidium bromide intercalation than is *P* (44). On intercalation, *P*_t_ was observed to decrease 3-fold (from ∼70 to ∼23 nm), while *P* changed <20%, reminiscent of our current results for chemical modification of the DNA grooves.

Finally, *γ*_0_ of the DNA-like polymers indicate adaptation to the various base modifications by under- or over-twisting relative to natural DNA (Figure 5D).

## DISCUSSION

### Mechanical properties are not predicted by bare polymer charge or base analog stacking

This work was undertaken to determine whether the mechanical properties of DNA-like polymers could be predictably manipulated by changing stacking energy and/or electrostatic tension. Such a result could give insight into the balance of forces operating within a DNA-like double helix and might prove useful in materials science and engineering applications of DNA analogs (45). Interestingly, we do not find a simple correlation between stacking propensity (38) of modified bases in these DNA-like polymers (Supplementary Data S2) and the measured mechanical parameters. Perhaps, this result should not be surprising because the contribution of stacking effects to overall DNA stiffness is not predicted from the nearest-neighbor stacking characteristics of the 10 natural DNA dimeric steps (7). More surprising, there is no correlation between 12–25% increase or decrease in the bare charge of the DNA-like polymers and their mechanical properties. To compare this result with a recent theoretical predication (11), Table 1 contrasts predicted and observed bending persistence lengths for these DNA-like polymers (Supplementary Data S4). The experimental results reveal much more subtle than predicted electrostatic effects on bending persistence length. Together with the large unpredicted impacts of groove modifications on DNA-like polymer *P*_t_, these results emphasize our inability to predict the mechanical properties of DNA-like polymers from first principles.

### Correlations between mechanical properties and helical structure

**Figure 6.**
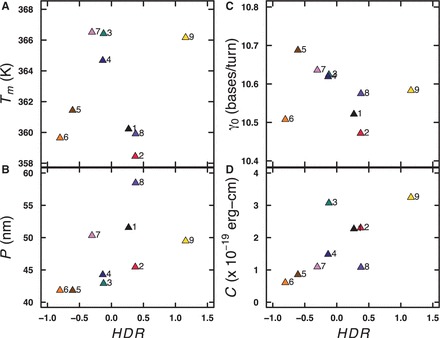
Relationship between experimentally determined parameters and *HDR*. *T*_m_ (**A**), *P* (**B**), *γ*_0_ (**C**) and *C* (**D**) are plotted as a function of *HDR*. See Figure 3 legend for details.

We were surprised and interested in the induced changes in DNA helical geometry suggested by the CD data (Figure 3). This raised the possibility that the dominant effect of analog substitution was to drive a transition in helical geometry resulting in DNA-like polymers with different mechanical properties. To test for general correlations between the DNA helical family and the experimentally calculated parameters of the DNA-like polymers, we plotted the various parameters as a function of the *HDR* (defined as the ratio of CD ellipticities at 290 and 201 nm). The results are shown in Figure 6. Perhaps not surprisingly, there is no quantitative correlation between this *HDR* and any single parameter, indicating that these parameters are likely influenced by a combination of factors. In particular, Figure 6 shows clearly that *T*_m_ and *γ*_0_ are poorly correlated with the *HDR*. Examining the plot of *P* in Figure 6B, there is a weak correlation between increasing *HDR* and *P*, noting that the overall changes in *P* (∼20%) are small. The plot of *C* in Figure 6D shows a stronger correlation between increasing *HDR* and increasing twist stiffness. Variant **9** with the most A-type character is the most resistant to twisting, and variant **6** with the most Z-like character is the least resistant to twisting. This trend generally holds true for the other variants as well. Taken together, these results suggest that incorporation of base analogs can change the helical conformation(s) of DNA-like polymers, and that these different conformations have different mechanical properties.

### Extended application of CC theory

An unexpected implication of this work is that Manning's classic polyelectrolyte CC theory (36) may have broader application than previously recognized. CC theory predicts that any polymer whose linear charge density exceeds a defined threshold value (∼1.4 charges/nm in monovalent salt) will spontaneously condense a compensating number of mobile counterions to achieve a net thermodynamic polymer charge that is equal for all such polymers *and independent of both the bare polymer charge density and the bulk salt concentration* (Supplementary Data S4). CC has long been known to predict the salt-dependence of charged ligand binding to DNA (46,47). Our current data suggest that the concept of CC is important for understanding electrophoretic mobility, ion exchange behavior, and *P* for DNA-like polymers. Our results suggest that it is largely the invariant *net* charge after CC that governs the behavior of DNA-like polymers. Thus, theories of electrophoresis, ion exchange chromatography and electrostatic tension in DNA-like polymers should consider in aggregate the polymers and their condensed counterions.

In contrast, our results do not support Manning’s recent ‘line of charge’ theory of DNA stiffness (11). We observe that changes in bare charge density do not predictably alter DNA-like polymer bend rigidity. This does not necessarily imply that electrostatic stretching forces do not contribute to stiffness. A constant electrostatic stretching contribution to polymer stiffness may arise from repulsive interactions between the *residual* charges on the polymer (constant and independent of the bare charge density so long as the bare charge density is above a threshold). This reasoning might explain the previous observation that a ‘meroduplex’ with half of DNA native charge is as stiff as natural DNA (20), as well as the previous observation that laterally asymmetric charge neutralization induces DNA bending (25).

We find that rather than modifying DNA stiffness through a mechanism easily interpretable as electrostatic, the more dominant effect of neutral and charged base modifications seems to be their ability to drive transitions to helical conformations different from canonical B-form DNA. These alternate conformations appear to be characterized especially by altered twist flexibility. Whether this altered twist flexibility of DNA-like polymers reflects a global transition in helical conformation, or the behavior of junctions between different conformational domains remains to be determined. If such junctions exist, they are not unpaired because the effects of the tested analogs on *T*_m_ and bend flexibility are subtle.

## SUPPLEMENTARY DATA

Supplementary Data are available at NAR Online, including [48–58].

## FUNDING

Mayo Graduate School; Mayo Foundation; National Institutes of Health (NIH) [GM075965 to L.J.M.; GM069773 to Y.T.]; Department of Science and Technology, India [SR/S1/OC-51/2009 to S.G.S. and S.P.Y.]. Funding for open access charge: Mayo Foundation; NIH.

*Conflict of interest statement*. None declared.

## Supplementary Material

Supplementary Data
