# Supplementary material for: Mechanical properties of DNA-like polymers
Source: Nucleic Acids Res. 2013 Sep 5;41(22):10593–604. doi: 10.1093/nar/gkt808 (PMC3905893; doi:10.1093/nar/gkt808)
Supplement: Supplementary Data [file supp_41_22_10593__index.html]

Mechanical properties of DNA-like polymers — Mechanical properties of DNA-like polymers — Supplementary Data 

# Mechanical properties of DNA-like polymers

## Supplementary Data

files

**Files in this Data Supplement:**

- Supplementary Data - pdf file
