# Supplementary material for: Mechanical properties of DNA-like polymers
Source: Nucleic Acids Res. 2013 Sep 5;41(22):10593–604. doi: 10.1093/nar/gkt808 (PMC3905893; doi:10.1093/nar/gkt808)
Supplement: Supplementary Data [file supp_gkt808_nar-01737-f-2013-File008.pdf]

Supplementary Data

Mechanical properties of DNA-like polymers

Justin P. Peters,[1] Shweta P. Yelgaonkar,[2] Seergazhi G. Srivatsan,[2] Yitzhak Tor,[3] and L. James Maher, III[1,*]

[1]Department of Biochemistry and Molecular Biology
Mayo Clinic College of Medicine
200 First St. SW, Rochester, MN 55905, USA
[2]Indian Institute of Science Education and Research
900, NCL Innovation Park, Dr. Homi Bhabha Road, Pune 411008, India
[3]Department of Chemistry and Biochemistry
University of California, San Diego
La Jolla, California 92093, USA
[*]Corresponding Author. E-mail: maher@mayo.edu.

S.1 Chemical Synthesis of Modified dUTPs S2
S.1.1 Materials and Instrumentation S3
S.1.2 Synthesis S3
S.2 Characterization of Modified dNTPs and Modified DNAs S5
S.2.1 Enzymatic Characterization of Modified DNAs S5
S.2.2 Liquid Chromatography/Mass Spectrometry (LC/MS) S8
S.2.3 Ion Exchange Chromatography S15
S.2.4 Dangling End Thermal Denaturation Experiments S16
S.2.5 Thermal Denaturation of Modified DNAs S19
S.2.6 Ultraviolet (UV) Absorption Spectroscopy S19
S.2.7 Circular Dichroism (CD) Spectroscopy S21
S.3 Cyclization Kinetics S23
S.3.1 Reaction Mechanism S23
S.3.2 *J*-factor Estimation S24
S.3.3 Increasing Mechanism Complexity S25
S.3.4 Assessing the Robustness of the Fitting Approach S26
S.3.5 Global Fitting S31
S.3.6 Cyclization Control Experiments S34
S.4 Manning Theory S37
S.4.1 Counterion Condensation (CC) Theory S37
S.4.2 Manning Theory of Persistence Length S38
References S39

S.1 Chemical Synthesis of Modified dUTPs

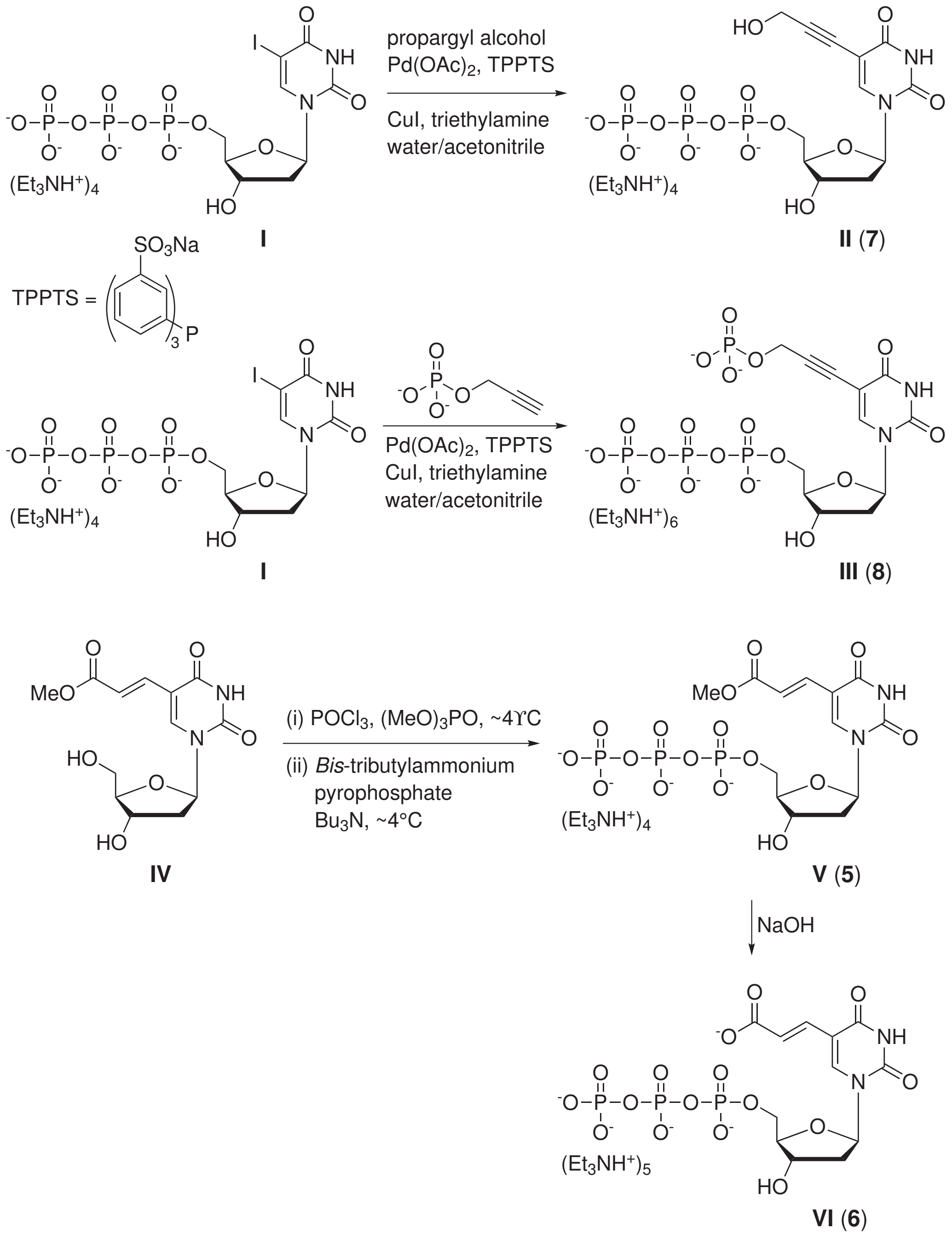

Figure S.1: Scheme for synthesis of phosphate- and carboxylate-modified dUTPs. The propargyl alcohol- and propargyl phosphate-modified dUTP analogs **II** and **III**, respectively, were synthesized (employing the aqueous-phase Sonogashira cross-coupling methodology (47)) by reacting 5-iodo-dUTP **I** with propargyl alcohol or propargyl phosphate in the presence of a water-soluble catalytic system, $Pd(OAc)_2$/TPPTS. The methyl ester- and carboxylate-modified dUTPs **V** and **VI**, respectively, were synthesized by following the reported procedure (35).

S.1.1 Materials and Instrumentation

5-iodo-2′-deoxyuridine, palladium acetate, trimethyl phosphate, propargyl alcohol, CuI and DEAE sephadex-A25 anion exchange resin were purchased from Sigma-Aldrich. TPPTS was obtained from Alfa Aesar and $POCl_3$ was obtained from Acros Organics. Milli-Q water (Millipore) was used in the preparation of buffers. NMR spectra were recorded on a Bruker Ultrashield 500 MHz WB Plus and Varian Mercury 400 MHz spectrometer. Mass measurements were recorded on an Applied Biosystems 4800 Plus MALDI TOF/TOF analyzer and a LCQDECA (Finnigan) ESI spectrometer with a quadrapole ion trap. Absorption spectra were recorded on a PerkinElmer Lambda 45 UV-Vis spectrophotometer.

S.1.2 Synthesis

5-Iodo-dUTP I (48)

To an ice cold solution of 5-iodo-2′-deoxyuridine (0.200 g, 0.56 mmol, 1.0 equiv) in trimethyl phosphate (2 mL) was added freshly distilled $POCl_3$ (130 μL, 1.42 mmol, 2.5 equiv). The solution was stirred for 28 h at 4–6 °C. TLC revealed completion of the reaction. A solution of *bis*-tributylammonium pyrophosphate (49) (0.5 M in DMF, 5.6 mL, 2.8 mmol, 5.0 equiv) and tri-*n*-butylamine (1.35 mL, 10.2 mmol, 10 equiv) was then rapidly added under ice-cold condition. The reaction was quenched after 30 min with 1 M triethylammonium bicarbonate buffer (TEAB, pH 7.5, 20 mL) and was extracted with ethyl acetate (20 mL). The aqueous layer was evaporated under vacuum. The residue was purified first on a DEAE sephadex-A25 anion exchange column (20–600 mM, TEAB buffer, pH 7.5). Fractions were analyzed by UV-Vis spectroscopy, and appropriate fractions were lyophilized to afford the desired triphosphate product **1** as a tetratriethyl ammonium salt (0.212 g, 38%). ^{1}H-NMR (400 MHz, D_2O): δ ppm 8.01 (s, 1H), 6.02 (t, J = 7.2 Hz, 1H), 4.41 (br, 1H), 3.98–3.94 (m, 3H), 2.14–2.12 (m, 2H); ^{31}P-NMR (162 MHz, D_2O): δ ppm -7.24 (br, P_γ), -10.71 (d, J = 20.6, P_α), -22.04 (br, P_β); ESI-MS (negative mode): Calculated for $C_9H_{14}IN_2O_{14}P_3$ [M] 593.87, found $[M–H]^-$ 592.89.

5-Propargyl alcohol-modified dUTP II

A degassed solution of water/acetonitrile (2:1, 1 mL) was added to a flask containing tetratriethyl ammonium salt of 5-Iodo-dUTP **I** (0.088 g, 0.088 mmol, 1 equiv), propargyl alcohol (16 μL, 0.27 mmol, 3.1 equiv) and CuI (0.003 g, 0.016 mmol, 0.18 equiv). A degassed solution of TEA (100 μL, 0.72 mmol, 8.2 equiv) was then added to the above solution. A degassed solution of $Pd(OAc)_2$ (0.0018 g, 0.0089 mmol, 0.09 equiv) and tri(3-sulfonatophenyl)phosphine hydrate sodium salt (TPPTS, 85%, 0.030 g, 0.046 mmol, 0.51 equiv) in 0.5 mL water/acetonitrile (2:1) was added slowly and the reaction mixture was heated at 60°C for 60 min. The reaction mixture was evaporated to dryness and was purified first on a DEAE sephadex-A25 anion exchange column (0.020–1.0 M, TEAB buffer, pH 7.5) followed by reverse-phase HPLC (Vydac C18 column, 1.0 × 25 cm, 5 μm TP silica, 0–11% acetonitrile in 100 mM triethyl ammonium acetate buffer, pH 7.0, 22 min). Appropriate fraction was lyophilized several times to afford the product **II** as a white foam (0.040 g, 48%). ^{1}H-NMR (500 MHz, D_2O): δ ppm 8.15 (s, 1H), 6.24 (t, J = 5.5 Hz, 1H), 4.59 (br, 1H), 4.37 (s, 2H), 4.16 (br, 3H), 2.35 (br, 2H); ^{13}C-NMR (125 MHz, D_2O): δ ppm 164.6, 150.5, 144.9, 99.1, 92.3, 85.7, 85.6, 75.9, 70.4, 65.2, 50.0, 38.8; ^{31}P-NMR (202 MHz, D_2O): δ ppm -11.22 (br, P_α, P_γ), -22.04 (br, P_β); ESI-MS (negative mode): Calculated for $C_{12}H_{17}N_2O_{15}P_3$ [M] 521.98, found $[M–H]^-$ 520.98.

5-Propargyl phosphate-modified dUTP III

A degassed solution of water/acetonitrile (2:1, 1 mL) was added to a flask containing tetratriethyl ammonium salt of 5-Iodo-dUTP **I** (0.076 g, 0.076 mmol, 1 equiv), propargyl phosphate-dicyclohexylammonium salt (50) (0.076 g, 0.23 mmol, 3.0 equiv) and CuI (0.003 g, 0.016 mmol, 0.21 equiv). A degassed sample of TEA (85 μL, 0.61 mmol, 8.0 equiv) was then added to the above solution. A degassed solution of $Pd(OAc)_2$ (0.002 g, 0.0089 mmol, 0.12 equiv) and TPPTS (85%, 0.031 g, 0.046 mmol, 0.61 equiv) in 0.5 mL water/acetonitrile (2:1) was added slowly and the reaction mixture was heated at 60°C for 60 min. The reaction mixture was evaporated to dryness and was purified first on a DEAE sephadex-A25 anion exchange column (0.020–1.0 M, TEAB buffer, pH 7.5) followed by reverse-phase HPLC (Vydac C18 column, 1.0 × 25 cm, 5 μm TP silica, 0–10% acetonitrile in 100 mM triethyl ammonium acetate buffer, pH 7.0, 20 min). Appropriate fraction was lyophilized several times to afford the product **III** as white foam (0.040 g, 43%). ^{1}H-NMR (500 MHz, D_2O): δ ppm 8.11 (s, 1H), 6.21 (t, J = 6.8 Hz, 1H), 4.65 (d, J = 8.05 Hz, 2H), 4.56 (br, 1H), 4.15 (br, 3H), 2.37–2.35 (m, 2H); ^{13}C-NMR (125 MHz, D_2O): δ ppm 164.6, 150.5, 144.9, 98.9, 90.6, 85.8, 85.0, 76.6, 70.5, 65.1, 54.0, 38.4; ^{31}P-NMR (202 MHz, D_2O): δ ppm 0.83 (propargyl P), -11.39 (br, P_α, P_γ), -23.20 (br, P_β); ESI-MS (negative mode): Calculated for $C_{12}H_{18}N_2O_{18}P_4$ [M] 601.95, found $[M–H]^-$ 600.95.

E-5-(2-methoxycarbonyl-vinyl)-dUTP V

The dUTP analog **V** was synthesized by following a reported procedure (35) To an ice cold solution of nucleoside **IV** (35) (0.10 g, 0.32 mmol, 1 equiv) in dry trimethyl phosphate (1.5 mL) was added freshly distilled $POCl_3$ (75 μL, 0.48 mmol, 2.5 equiv). The solution was stirred for 24 h at ∼4°C. A solution of *bis*-tributylammonium pyrophosphate (49) (0.5 M in DMF, 3.2 mL, 5 equiv) and tributylamine (0.76 mL, 3.2 mmole, 10 equiv) was simultaneously added under ice cold condition. The reaction was quenched after 20 min with TEAB buffer (1 M, pH 7.5, 20 mL) and was extracted with ethyl acetate (20 mL). Aqueous layer was evaporated and the residue was purified first on a DEAE Sephadex-A25 anion exchange column (10 mM–1M TEAB buffer, pH 7.5) followed by reverse-phase flash column chromatography (C18 RediSepRf, 0–40% acetonitrile in 100 mM triethyl ammonium acetate buffer, pH 7.2, 40 min). Appropriate fractions were lyophilized to afford the desired triphosphate product **V** as a tetratriethylammonium salt (0.039 g, 13%). ^{1}H-NMR (500 MHz, D_2O): δ ppm 8.15 (s, 1H), 7.44 (d, J = 16.2 Hz. 1H), 6.84 (d, J = 15.9 Hz, 1H), 6.27 (t, J = 6.7 Hz, 1H), 4.63 (br, 1H), 4.20 (br, 3H), 3.76 (s, 3H), 2.41−2.39 (m, 2H); ^{31}P-NMR (202.5 MHz, D_2O): δ ppm -10.82 (br), -11.58 (br), -23.26 (br); MALDI-TOF MS (negative mode): Calculated for $C_{13}H_{19}N_2O_{16}P_3$ [M] 551.99, found $[M–H]^-$ 550.88.

E-5-(2-carboxy-vinyl)-dUTP VI

The dUTP analog **VI** was synthesized by following a reported procedure (35). dUTP **V** (0.026 g, 27 μmol) was dissolved in 2 mL of water and an aqueous solution of NaOH (0.4 M, 2 mL) was added to the above solution. The reaction mixture was stirred for 45 min at room temperature and purified by reverse-phase flash column chromatography (C18 RediSepRf, 0−40% acetonitrile in 100 mM triethyl ammonium acetate buffer, pH 7.2, 40 min) to afford product **VI** (0.025 g, 89%). ^{1}H-NMR (500 MHz, D_2O): δ ppm 8.02 (s, 1H), 7.10 (d, J = 15.9 Hz, 1H), 6.79 (d, J = 15.9 Hz, 1H), 6.28 (t, J = 6.4 Hz, 1H), 4.63 (br, 1H), 4.20 (br, 3H), 2.49−2.37 (m, 2H); ^{31}P-NMR (202.5 MHz, D_2O): δ ppm -10.26 (br), -11.41 (br), -22.98 (br); MALDI-TOF MS (negative mode): Calculated for $C_{12}H_{17}N_2O_{16}P_3$ [M] 537.98, found $[M–H]^-$ 536.88.

S.2 Characterization of Modified dNTPs and Modified DNAs

S.2.1 Enzymatic Characterization of Modified DNAs

Cloned intrinsically straight ∼200-bp sequences were created using unique 5-bp direct repeats to eliminate long-range sequence-directed curvature (26) and were the kind gift of A. Vologodskii. The longest (parent) construct in this series was designated pJ823 and is shown in Figure S.2.

```
  1  GGGTAACGCC AGGGTTTTCC CAGTCACGAC GTTGTAAAAC GACGGCCAGT GAATTCGAGC TCGGTACCCG GGGATCCTCT
     CCCATTGCGG TCCCAAAAGG GTCAGTGCTG CAACATTTTG CTGCCGGTCA CTTAAGCTCG AGCCATGGGC CCCTAGGAGA

 81  AGAAGCTTAC TCGACTCGAG CCTAGCCTAT GACATGACAC GTTACGTTAG TCGAGTCGAT CAGATCAGAC GCTACGCTAG
     TCTTCGAATG AGCTGAGCTC GGATCGGATA CTGTACTGTG CAATGCAATC AGCTCAGCTA GTCTAGTCTG CGATGCGATC

161  CTGAGCTGAC TGTACTGTAT GCAATGCAAC CTCACCTCAG GACAGGACAC GTGACGTGAT GCTATGCTAC CAGACCAGCT
     GACTCGACTG ACATGACATA CGTTACGTTG GAGTGGAGTC CTGTCCTGTG CACTGCACTA CGATACGATG GTCTGGTCGA

241  GCACTGCAGA CTGGACTGAC GCTACGCTAT CGCATCGCAG ATGAGATGAA GCTTGGCGTA ATCATGGTCA TAGCTGTTTC
     CGTGACGTCT GACCTGACTG CGATGCGATA GCGTAGCGTC TACTCTACTT CGAACCGCAT TAGTACCAGT ATCGACAAAG

321  CTGTGTGAAA TTGTTATCCG CTCACAATTC CACACAACAT ACGAGCCGGA AGCATAAAGT GTAAAGCCTG GGGTGCCTAA
     GACACACTTT AACAATAGGC GAGTGTTAAG GTGTGTTGTA TGCTCGGCCT TCGTATTTCA CATTTCGGAC CCCACGGATT

401  TGAGTGAGCT AACTCACA
     ACTCACTCGA TTGAGTGT
```

(a) Plasmid pJ823

```
  1  GGGTAACGCC AGGGTTTTCC CAGTCACGAC GTTGTAAAAC GACGGCCAGT GAATTCGAGC TCGGTACCCG GGGATCCTCT
     CCCATTGCGG TCCCAAAAGG GTCAGTGCTG CAACATTTTG CTGCCGGTCA CTTAAGCTCG AGCCATGGGC CCCTAGGAGA

 81  CGCGGCGCCC GCGACTCGAG CCTAGCCTAT GACATGACAC GTTACGTTAG TCGAGTCGAT CAGATCAGAC GCTACGCTAG
     GCGCCGCGGG CGCTGAGCTC GGATCGGATA CTGTACTGTG CAATGCAATC AGCTCAGCTA GTCTAGTCTG CGATGCGATC

161  CTGAGCTGAC TGTACTGTAT GCAATGCAAC CTCACCTCAG GACAGGACAC GTGACGTGAT GCTATGCTAC CAGACCAGCT
     GACTCGACTG ACATGACATA CGTTACGTTG GAGTGGAGTC CTGTCCTGTG CACTGCACTA CGATACGATG GTCTGGTCGA

                                              *9 87654321
241  GCACTGCAGA CTGGACTGAC GCTACGCTAT CGCATCGCAG ATGAGATGAA GCCGGGCGCC GCCATGGTCA TAGCTGTTTC
     CGTGACGTCT GACCTGACTG CGATGCGATA GCGTAGCGTC TACTCTACTT CGGCCCGCGG CGGTACCAGT ATCGACAAAG

321  CTGTGTGAAA TTGTTATCCG CTCACAATTC CACACAACAT ACGAGCCGGA AGCATAAAGT GTAAAGCCTG GGGTGCCTAA
     GACACACTTT AACAATAGGC GAGTGTTAAG GTGTGTTGTA TGCTCGGCCT TCGTATTTCA CATTTCGGAC CCCACGGATT

401  TGAGTGAGCT AACTCACA
     ACTCACTCGA TTGAGTGT
```

(b) Plasmid pJ1506

Figure S.2: Template DNA sequences. Forward (LJM-3222, magenta) and reverse (LJM-3223, green) primers are indicated along with two distinct restriction sites (cyan and orange, bold). Red indicates sequence differences (sites of mutagenesis) between the two parent plasmids. The * symbol (value 10) and numbers (X = 9,8,. . . ,1) designate plasmid pJ1750 and the plasmid series pJ174X, respectively, as well as the length of the deletion from the parent plasmid.

Site-directed and deletional mutageneses were performed using the QuikChange Lightning Kit following the protocol recommended by the supplier (Stratagene) to generate plasmid pJ1506 and the new series pJ1741 – pJ1750, each differing in length by one base pair (Figure S.2). Using the parent plasmids as templates for PCR yields ∼400-bp PCR products. The pJ823 PCR product is cleaved by *Hin*dIII digestion (recognition sequence 5′-AAGCTT) into three easily distinguishable

products, Figure S.3(a). The mutagenized template pJ1506 results in a PCR product that can be cleaved into three products by a variety of restriction enzymes which recognize the recognition sequence 5′-GGCGCC, such as *Kas*I, Figure S.3(b), or *Nar*I, Figure S.3(c).

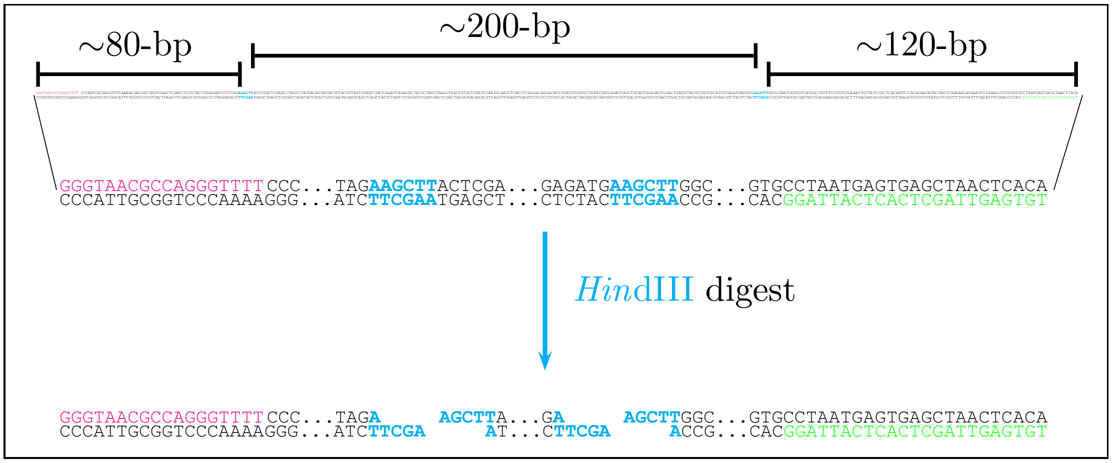

(a) *Hind*III

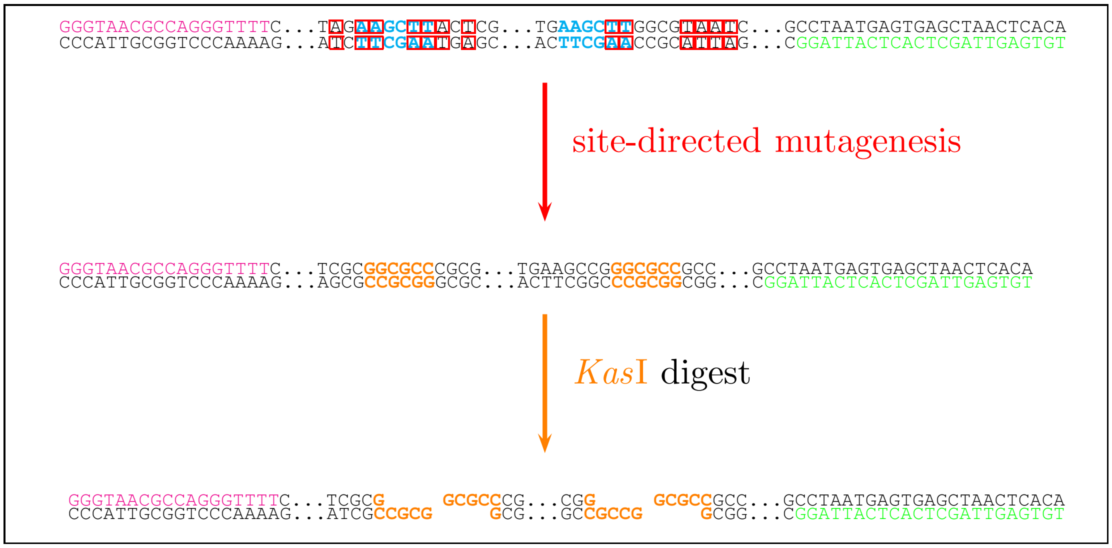

(b) *Kas*I

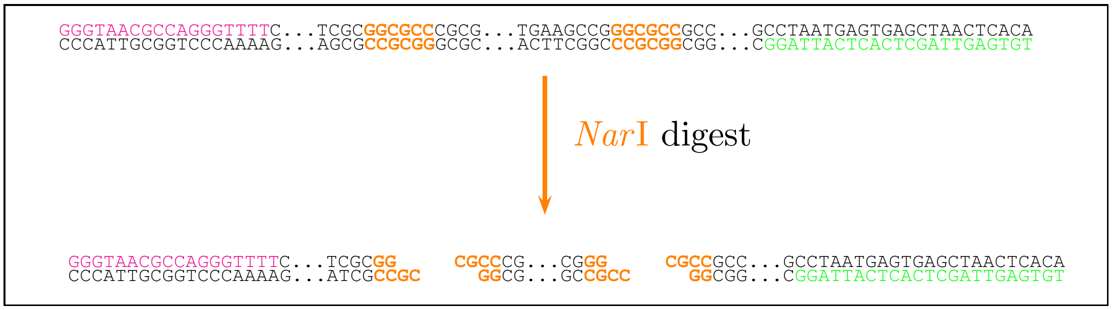

(c) *Nar*I

Figure S.3: Comparison of Restriction Endonuclease Digestion

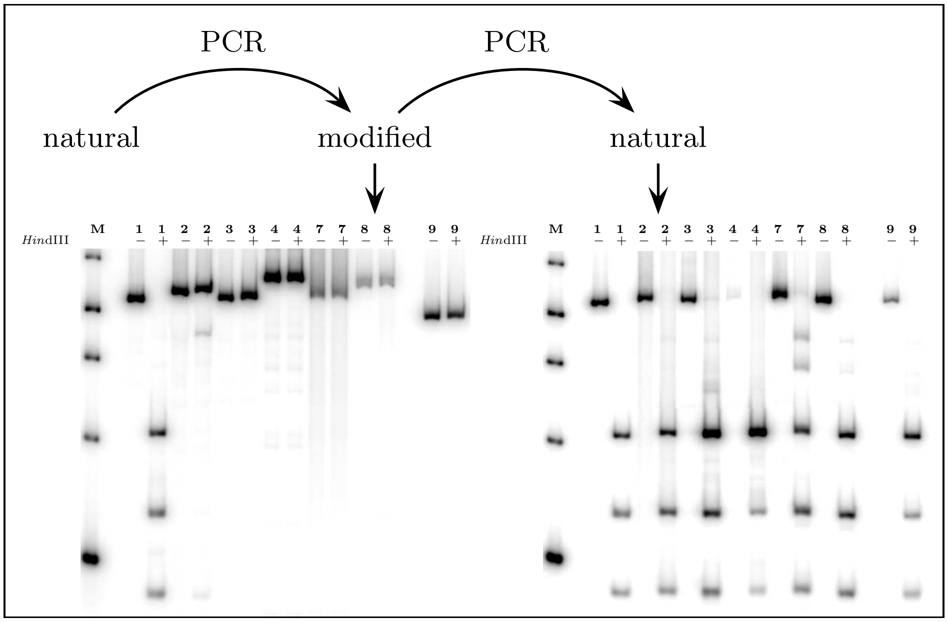

(a) pJ823 Template

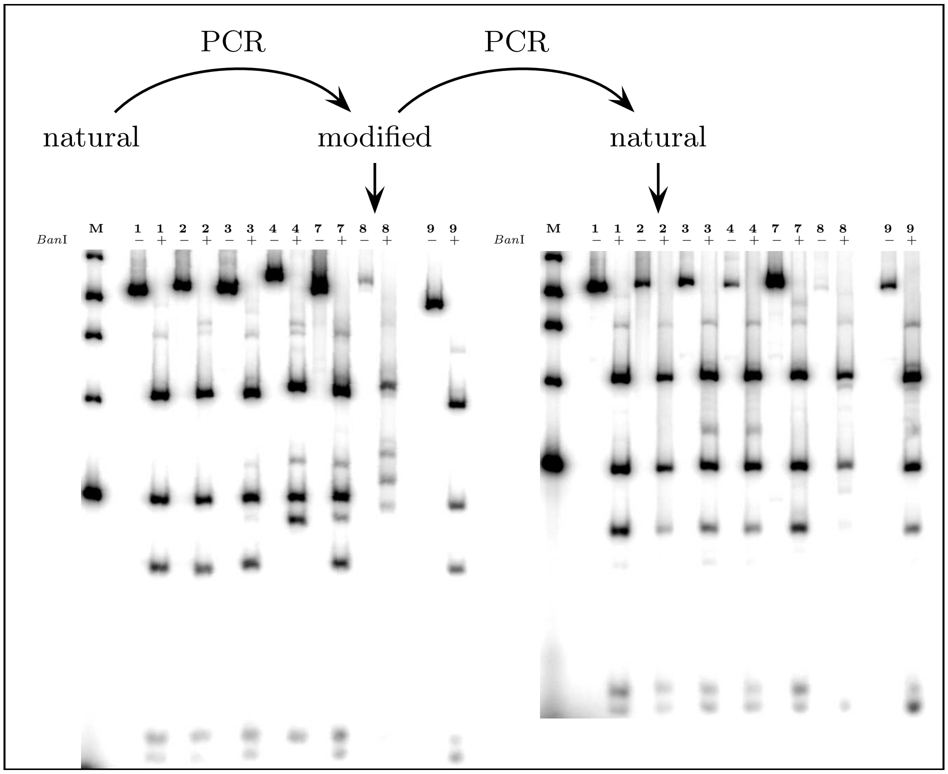

(b) pJ1506 Template

Figure S.4: Restriction Endonuclease Susceptibility of Modified DNAs.

Figure S.4 shows the restriction endonuclease susceptibility of the modified DNAs. There is clear *Hin*dIII restriction inhibition when plasmid pJ823 is the DNA template. This behavior is not unexpected as it has been previously demonstrated that modified bases can impact the enzymatic

activities of type II restriction endonucleases (51). In this study, the rates of cleavage of DNAs with substituents at the 5 position of dT were measured to be slower for a variety of sequence-specific restriction endonucleases with A·T base pairs (e.g. *Hin*dIII, recognition sequence 5′-AAGCTT) while unaffected for G·C base pairs (e.g. *Hha*I, recognition sequence 5′-GCGC). However, the presence or absence of an A·T base pair was not a strict delimiter, suggesting that specific contacts of a given enzyme with an A·T base pair are crucial. Importantly, as shown in Figure S.4(a), when the modified DNA is reverted to natural DNA, the restriction block is alleviated. This restriction rescue demonstrates that mutagenesis and base mispairing are low.

In order to generate probes for cyclization, it is necessary to have robust and faithful cleavage of the modified DNAs. The new template pJ1506 and its truncation series (Figure S.2) were generated with this consideration in mind. The restriction site for release of the cyclization probes (5′-GGCGCC) was intentionally engineered to be devoid of A·T base pairs. Since only dT and dA residues are being modified in these experiments, restriction digest at these sites of DNA template pJ1506 is unaffected, Figure S.4(b). For technical reasons, *Ban*I (recognition sequence 5′-GGYRCC where Y is any pyrimidine and R is any purine) was utilized in place of *Kas*I (recognition sequence 5′-GGCGCC), with both enzymes producing 4 base 5′ overhangs. This switch introduced two new restriction sites so that the ∼80-bp and ∼120-bp products were further cleaved to produce ∼20-bp fragments. However, the ∼200-bp product (of importance) is unaffected. Finally, reverting to natural DNA allows for efficient restriction digest, Figure S.4(b).

S.2.2 Liquid Chromatography/Mass Spectrometry (LC/MS)

High-performance liquid chromatography (HPLC)-grade water and methanol were purchased from Burdick and Jackson. Analytes were separated using an HPLC system (Agilent series 1100, Agilent Technologies) equipped with a vacuum degasser, an autosampler, and a reverse-phase C18 analytical column (Phenomenex-C18 1.0×250 mm). The column was utilized at room temperature (∼ 23°C) and absorbance was monitored at 260 nm with a UV detector. The makeup of the LC mobile phases was water (mobile phase A) and methanol (mobile phase B). Separation was achieved by using the following elution conditions: 0 − 3 min, 2% B isocratic; 3 − 22 min, linear gradient 2 − 20% B; 22 − 35 min, 20% B isocratic; and, finally, reconditioning steps of the column were 2% B isocratic for 10 min after washing the column for 5 min using 95% B (52). The flow rate was 0.06 mL/min, and 8 μL injections were made of the standards and samples in mobile phase A. The HPLC system was connected to a time-of-flight mass spectrometer (MSD-TOF, Agilent Technologies) equipped with an electrospray interface. The instrument was operated in positive mode under the following operating parameters: capillary 4,000 V; nebulizer 20 psi; drying gas 7 L/min; gas temperature 325°C; fragmentor 45 V; skimmer 60 V; Oct 1 DC 37.5 V; Oct RF 250 V. The instrument was calibrated using the calibrant mixture provided by the manufacturer over the 50–3200 *m/z* range. The scan for data acquisition was the 100–500 *m/z* range. Agilent MassHunter Quantitative Analysis software was used for analysis.

Enzymatic Digestion

Modified dNTPs were dephosphorylated to mononucleosides using Antarctic phosphatase and the protocol suggested by the supplier (New England Biolabs, NEB). Alkaline phosphatase was not used because of reports of contamination with deoxyadenosine deaminase activity (53). Modified DNAs were digested to mononucleosides with a cocktail mix containing deoxyribonuclease I (DNase I), micrococcal nuclease (MNase), snake venom phosphodiesterase (SVPD) and Antarctic phosphatase (AP). Reactions (40 μL) contained ∼1 μg modified DNA, micrococcal nuclease buffer

(NEB) supplemented with 10 mM $MgCl_2$ and 0.1 mM $ZnCl_2$, 6 U DNase I (NEB), 20 U MNase (NEB), 2 U SVPD (Worthington Biochemical), and 5 U AP (NEB). Digestion occurred overnight at 37°C.

Modified Nucleosides

LC/MS analysis was performed using nucleosides derived from the modified dNTPs, presented in Figure S.5, Figure S.6, and Figure S.7. Retention times from HPLC chromatography were a sufficient discriminator of the various modifications (Table S.1). It should be noted that nucleosides **7** and **8** behaved identically, presumably due to conversion (dephosphorylation) of nucleoside **8** to nucleoside **7**. The suspicion that Antarctic Phosphatase removed the additional phosphate group from nucleoside **8** was further confirmed by examining the product-ion spectra of the modified nucleosides (Figure S.7 and Table S.2).

Nucleoside	retention time ± s.d. (min)	# runs
dA	26.45 ± 0.37	6
dC	9.37 ± 0.44	6
dG	19.58 ± 0.39	6
dT	21.39 ± 0.14	5
**2**	13.83 ± 0.40	7
**3**	29.52 ± 0.35	6
**4**	19.10 ± 2.66	12
**5**	41.30 ± 0.15	9
**6**	14.93 ± 0.25	6
**7**	23.93 ± 0.40	8
**8**	24.03 ± 0.35	7
**9**	24.81 ± 0.37	6

Table S.1: Retention times of nucleosides

Nucleoside	Ion	Formula (M)	Meas. *m/z*	Calc. *m/z*	Diff. (ppm)
dA	$[M+H]^+$	$C_{10}H_{13}N_5O_3$	252.1095	252.1091	-1.48
dC	$[M+H]^+$	$C_9H_{13}N_3O_4$	228.0980	228.0979	-0.73
dG	$[M+H]^+$	$C_{10}H_{13}N_5O_4$	268.1036	268.1040	1.63
dT	$[M+H]^+$	$C_{10}H_{14}N_2O_5$	243.0970	243.0975	2.37
**2**	$[M+H]^+$	$C_9H_{12}N_2O_5$	229.0817	229.0819	0.65
**3**	$[M+H]^+$	$C_{12}H_{14}N_2O_5$	267.0971	267.0975	1.63
**4**	$[M+H]^+$	$C_{12}H_{17}N_3O_5$	284.1259	284.1241	-6.50
**5**	$[M+H]^+$	$C_{13}H_{16}N_2O_7$	313.1033	313.1030	-0.77
**6**	$[M+H]^+$	$C_{12}H_{14}N_2O_7$	299.0872	299.0874	0.52
**7**	$[M+H]^+$	$C_{12}H_{14}N_2O_6$	283.0932	283.0925	-2.68
**8**	$[M+H]^+$	$C_{12}H_{14}N_2O_6$	283.0936	283.0925	-4.13
**9**	$[M+H]^+$	$C_{10}H_{14}N_6O_3$	267.1213	267.1200	-4.63

Table S.2: Summary of product ions corresponding to nucleosides. Ions, formulas, the calculated (Calc.) and measured (Meas.) mass-to-charge ratios (*m/z*), and differences (Diff.) thereof in parts per million (ppm) relating to Figure S.7.

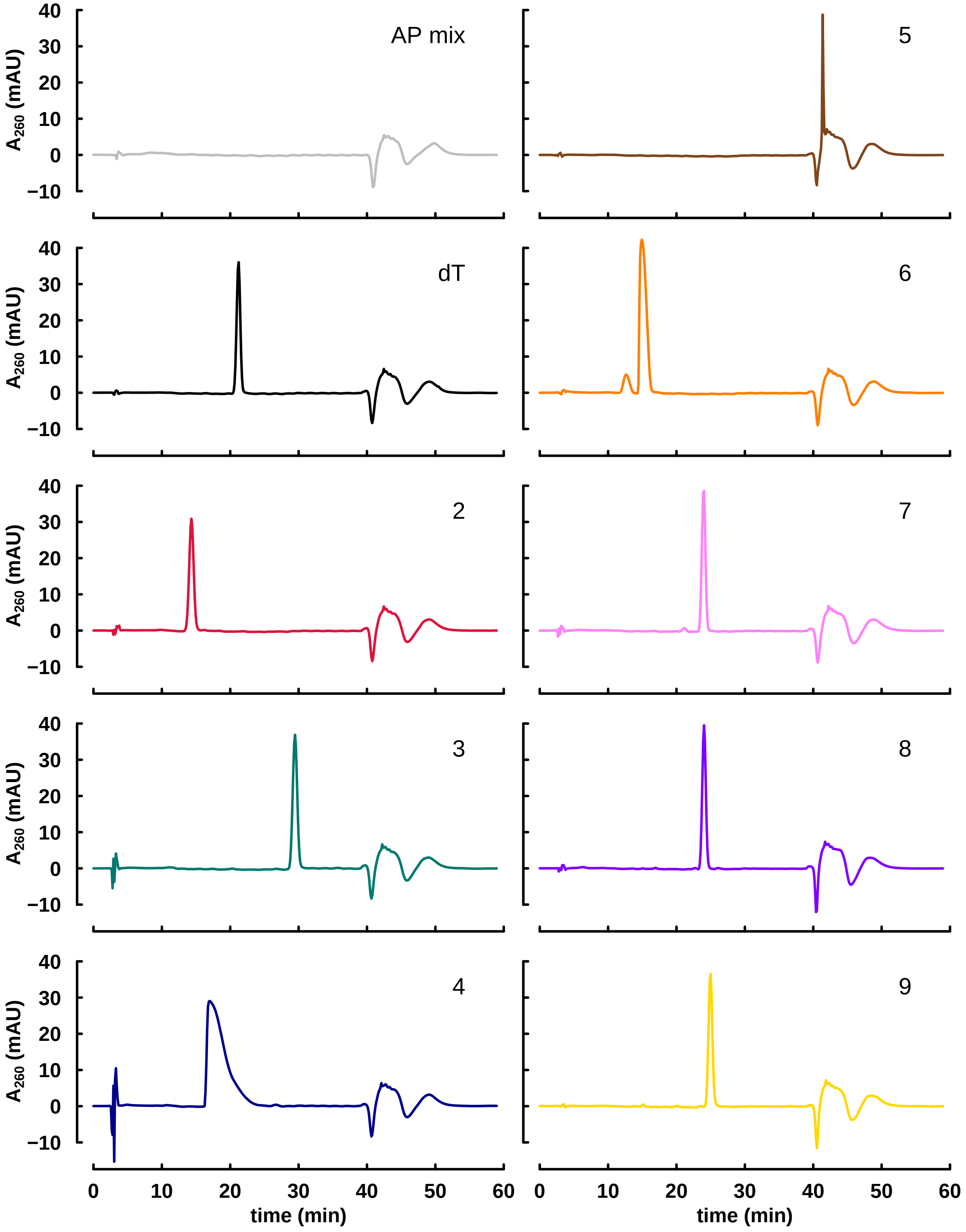

Figure S.5: Elution profiles of modified nucleosides.

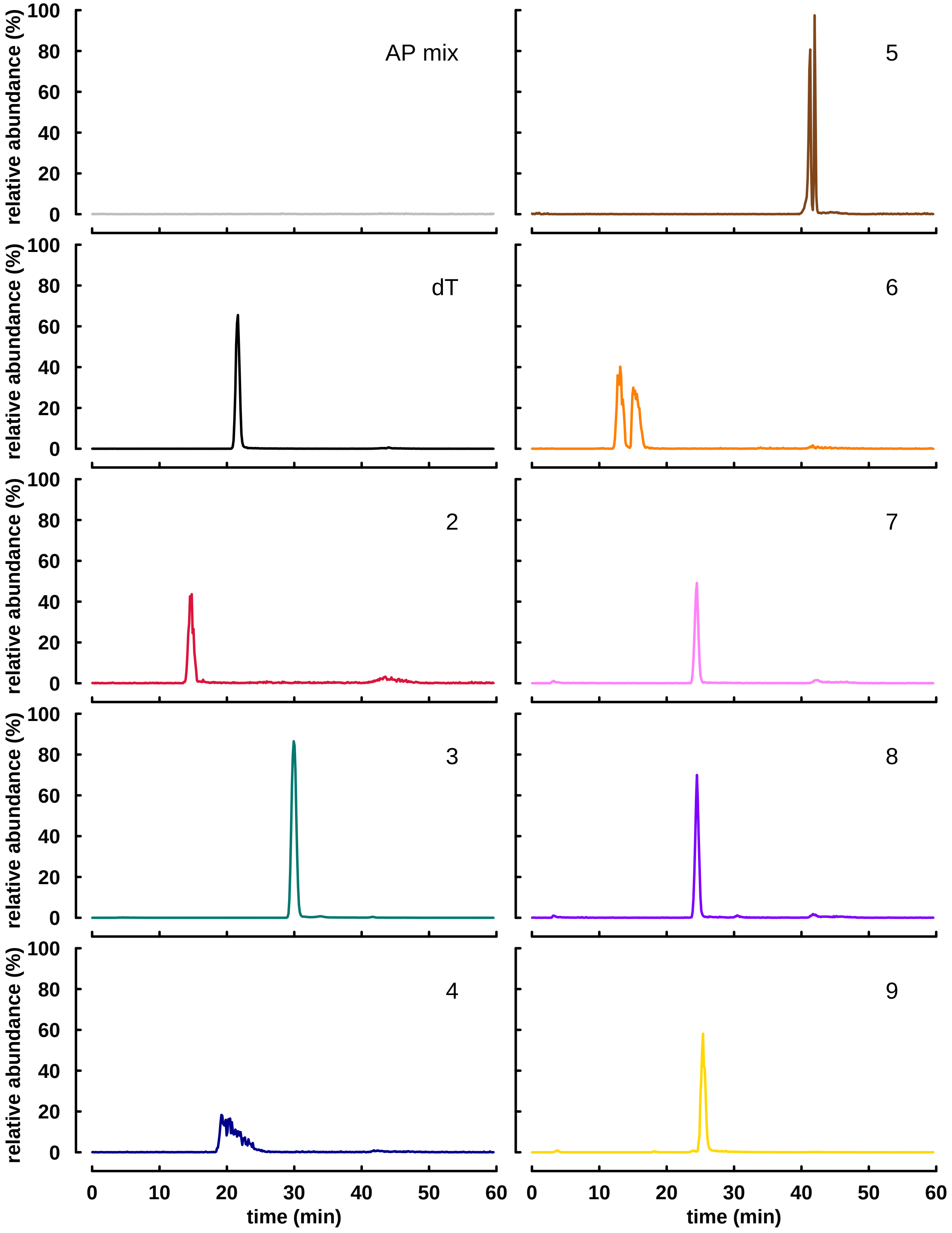

Figure S.6: Extracted ion chromatograms (EIC) of modified nucleosides.

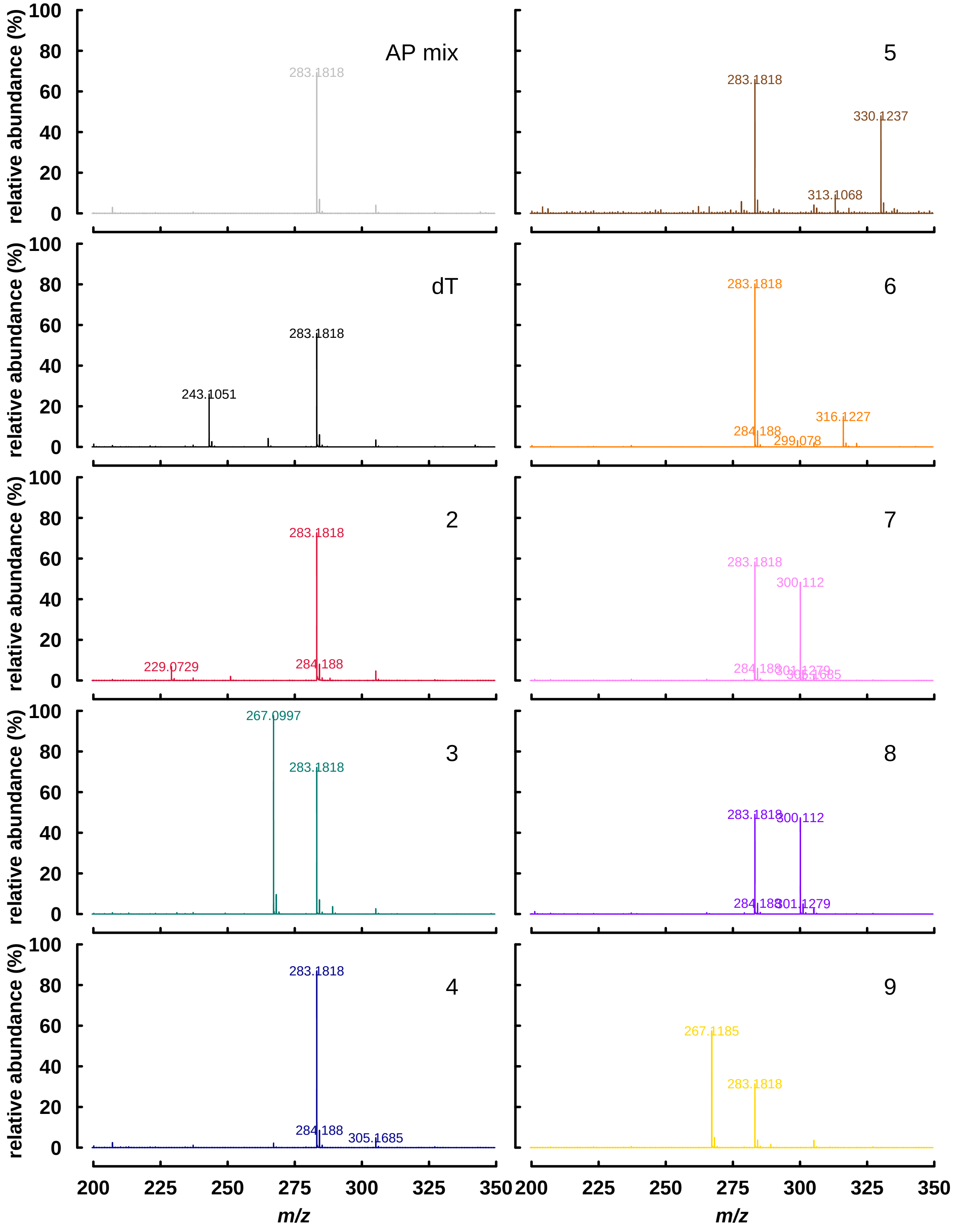

Figure S.7: Mass spectra of modified nucleosides (at retention peaks).

Modified DNAs

LC/MS analysis was performed using nucleosides derived from enzymatic digestion of the modified DNAs. Representative HPLC chromatograms are presented in Figure S.8. Analysis was also performed using DNAs modified on only one strand which could be produced in greater abundance. This was especially important for the variants with additional charge, DNAs **4** and **6**, which exhibited poorer separation on the reverse-phase column. The relevant fragment ions produced from degradation of the modified DNAs are listed in Table S.3.

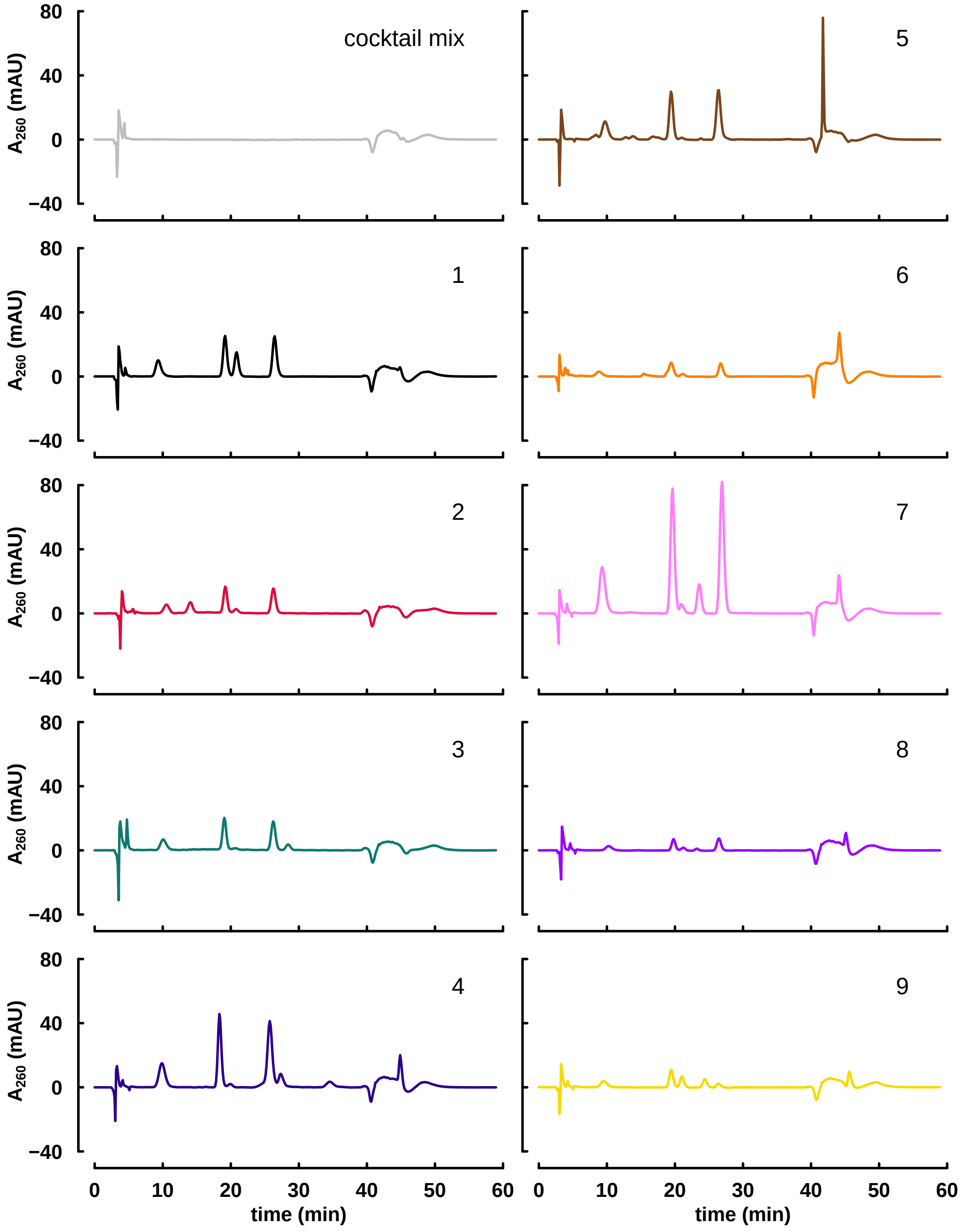

Figure S.8: Elution profiles of modified DNA digests.

DNA	Ion	Formula (M)	Meas. m/z	Calc. m/z	Diff. (ppm)
dA	$[M+H]^+$	$C_{10}H_{13}N_5O_3$		252.1091	
**1**			252.1103		-4.53
**2**			252.1097		-2.46
**3**			252.1097		-2.28
**4**			252.1102		-4.31
**5**			252.1103		-4.56
**6**			252.1103		-4.85
**7**			252.1103		-4.62
**8**			252.1103		-4.88
**9**			252.1109		-6.92
dC	$[M+H]^+$	$C_9H_{13}N_3O_4$		228.0979	
**1**			228.0994		-6.72
**2**			228.0986		-3.19
**3**			228.0975		1.59
**4**			228.0990		-5.08
**5**			228.0991		-5.30
**6**			228.0993		-6.30
**7**			228.0992		-5.94
**8**			228.0989		-4.62
**9**			228.0996		-7.68
dG	$[M+H]^+$	$C_{10}H_{13}N_5O_4$		268.1040	
**1**			268.1045		-1.85
**2**			268.1037		1.39
**3**			268.1032		3.01
**4**			268.1046		-2.22
**5**			268.1047		-2.52
**6**			268.1040		0.20
**7**			268.1048		-2.94
**8**			268.1037		1.19
**9**			268.1045		-1.79
dT	$[M+H]^+$	$C_{10}H_{14}N_2O_5$		243.0975	
**1**			243.0988		-5.24
**2**			243.0977		-0.83
**3**			243.0990		-5.84
**4**			243.0988		-4.98
**5**			243.0985		-3.87
**6**			243.0973		1.04
**7**			243.0963		5.20
**8**			243.0951		10.12
**9**			243.0988		-5.26
**2**	$[M+H]^+$	$C_9H_{12}N_2O_5$	229.0808	229.0819	4.97
**3**	$[M+H]^+$	$C_{12}H_{14}N_2O_5$	267.0972	267.0975	1.16
**4**	$[M+H]^+$	$C_{12}H_{17}N_3O_5$	284.1259	284.1241	-6.50
**5**	$[M+H]^+$	$C_{13}H_{16}N_2O_7$	313.1054	313.1030	-7.61
**6**	$[M+H]^+$	$C_{12}H_{14}N_2O_7$	299.0868	299.0874	2.06
**7**	$[M+H]^+$	$C_{12}H_{14}N_2O_6$	283.0908	283.0925	5.86
**8**	$[M+H]^+$	$C_{12}H_{14}N_2O_6$	283.0908	283.0925	5.86
**9**	$[M+H]^+$	$C_{10}H_{14}N_6O_3$	267.1204	267.1200	-1.44

Table S.3: Summary of fragment ions produced from digests of modified DNAs.

S.2.3 Ion Exchange Chromatography

Anion exchange chromatography was performed on 98-bp modified DNAs. The elution profiles (monitored by absorbance at 260 nm) of the modified DNAs are shown in Figure S.9. All of the modified DNAs eluted between 14 and 20 minutes. From the elution profiles of independent runs, retention times (and standard deviations) were tabulated. The retention time differences from unmodified DNA **1** are listed in Table S.4. No simple correlation was discernible between retention and analog charge.

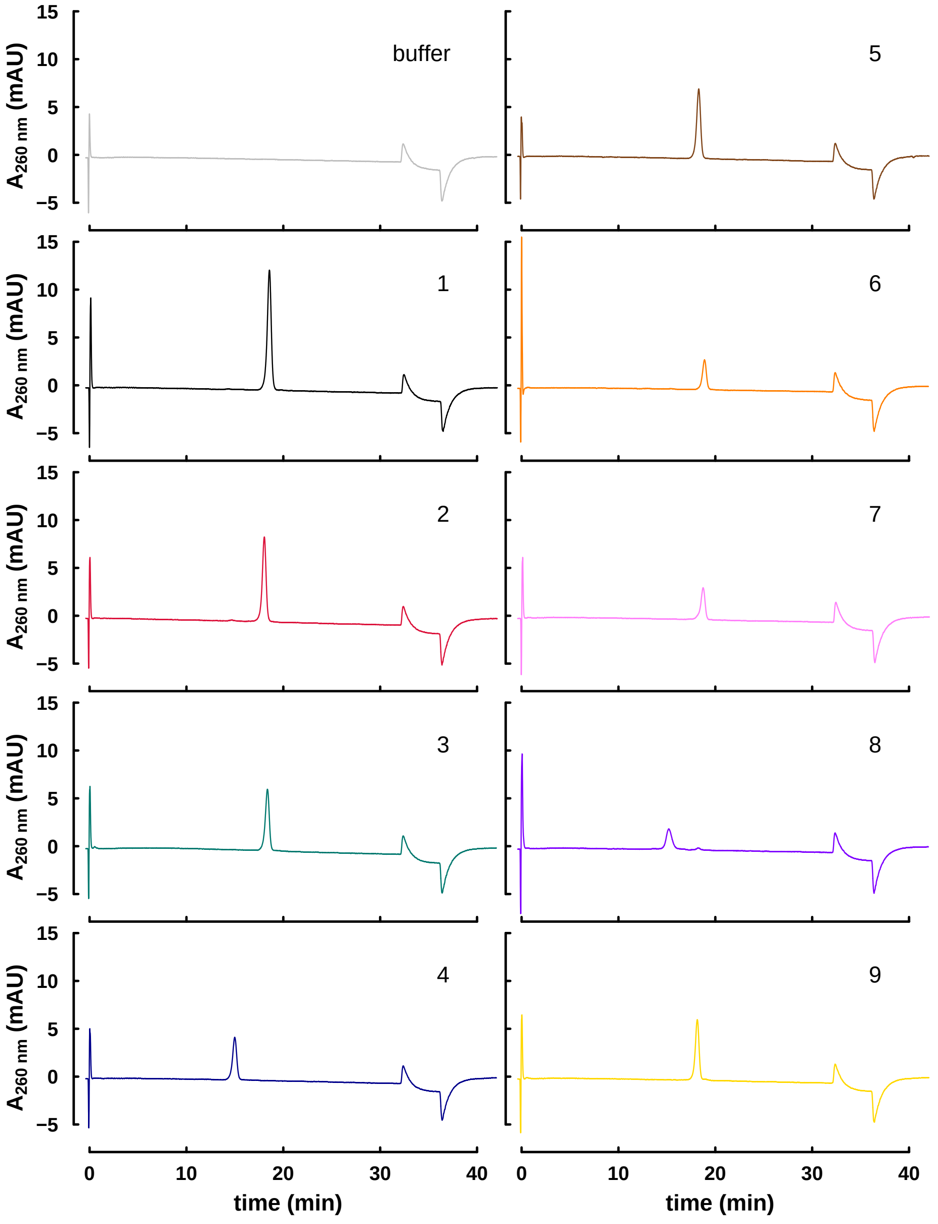

Figure S.9: Anion exchange chromatograms of modified DNAs.

DNA	time difference (s)	DNA	time difference (s)	DNA	time difference (s)
**1**	0 ± 1.7	**4**	-213.2 ± 1.6	**7**	8.9 ± 3.7
**2**	-34.3 ± 5.7	**5**	-16.4 ± 1.6	**8**	-198.7 ± 2.5
**3**	-13.8 ± 3.8	**6**	17.4 ± 3.8	**9**	-24.2 ± 0.1

Table S.4: Difference in retention time from natural DNA.

S.2.4 Dangling End Thermal Denaturation Experiments

To determine the base stacking propensities of the analogs, "dangling end" thermal denaturation experiments (37) were performed for various unpaired nucleotides (X) in the self-complementary DNA oligonucleotide 5′-XCGCGCG. The core oligonucleotide (lacking X) as well as oligonucleotides with X = A, C, G, T, and 2′-deoxyuridine (**2**) were purchased from Integrated DNA Technologies. Oligonucleotides with X = 5-propynyl-2′-deoxyuridine (**3**), 5-aminoallyl-2′-deoxyuridine (**4**), 5-(carboxy)vinyl-2′-deoxyuridine (**6**), and 2,6-diaminopurine-2′-deoxyriboside (**9**) were purchased from TriLink BioTechnologies. Experiments (similar to those described in the main text) were performed over a temperature range of 20–80°C, measurements collected every 0.1°C with a temperature slope of 24°C/h. Samples (20 μL) contained 300–900 ng DNA ($\sim$5–25 μM as determined from nearest neighbor methods (28) using the extinction coefficients given in subsection S.2.6) in 10 mM sodium phosphate, pH 7.0 with 1 M NaCl and either 0.05X, 0.1X, 0.15X, or 0.2X SYBR Green I (Invitrogen).

Thermodynamic parameters were determined from a variation of the van't Hoff equation. From the definition of Gibbs free energy

$$\Delta G^\circ = \Delta H^\circ - T\Delta S^\circ \tag{S.1}$$

and the well known equation relating Gibbs free energy to the equilibrium constant K_a

$$\Delta G^\circ = -RT\ln(K_a) \tag{S.2}$$

it is easy to derive the classic form of the van't Hoff equation

$$\ln(K_a) = -\frac{\Delta H^\circ}{R}\left(\frac{1}{T}\right) + \frac{\Delta S^\circ}{R} \tag{S.3}$$

For the bimolecular reaction of a self-complementary oligo ($2S \rightleftharpoons D$), the total strand concentration (C_t) is constant (and equal to the starting concentration of the DNA oligo)

$$C_t = [S] + 2[D] \tag{S.4}$$

The fraction folded (or duplex strand proportion of total strands) is

$$\theta = \frac{2[D]}{C_t} \tag{S.5}$$

so that the equilibrium constant is

$$K_a = \frac{[D]}{[S]^2} = \frac{\theta}{2C_t(1-\theta)^2} \tag{S.6}$$

At the T_m it follows that $[S] = C_t/2$ and $[D] = C_t/4$, so that $K_a = C_t^{-1}$. These expressions are used to derive an alternative form of the van't Hoff equation

$$\frac{1}{T_m} = \frac{R}{\Delta H^\circ}\ln C_t + \frac{\Delta S^\circ}{\Delta H^\circ} \tag{S.7}$$

whose slope $R/\Delta H^\circ$ and intercept $\Delta S^\circ/\Delta H^\circ$ give the desired parameters. This analysis assumes that the plot is absolutely linear, and that ΔH° and ΔS° are temperature independent over the T_m range. It is further assumed that there is no change in heat capacity for the melting transition, i.e. $\Delta C_p = 0$. It has been shown that slight curvature in van't Hoff plots due to $\Delta C_p \neq 0$ can lead to significant errors in graphically evaluated thermodynamic parameters (54). However, since the enthalpies and entropies derived in this graphical manner are correlated, the relative error of ΔG° is typically smaller than the relative errors of ΔH° and ΔS°.

To obtain T_m data as a function of concentration (C_t), the thermal denaturation data were processed following a two-state transition model (27). The two states are referred to as "folded" (fully associated) and "unfolded" (fully dissociated) for simplicity. The data were converted from fluorescence (F) as a function of temperature (T) to fraction folded (θ) as a function of temperature

$$\theta(T) = \frac{F(T) - u(T)}{f(T) - u(T)} \tag{S.8}$$

where the unfolded (u) and folded (f) baselines were determined from the data by linear regression. Within the analysis interval ($0.15 < \theta < 0.85$) the midpoint transition temperature (T_m) was evaluated from $\theta = 0.5$.

The results for the natural DNA nucleotides (and core sequence) are presented in Table S.5 and Figure S.10. Stabilization of the core sequence duplex from base stacking is reported as both ΔT_m and $\Delta\Delta G^\circ$. All of the tested analogs stabilized the core hexamer duplex, with a maximal increase in T_m of 11.8°C and most favorable free energy of stacking ($\Delta\Delta G^\circ_{37}$) of 1.7 kcal/mol. The stacking order of the four natural DNA nucleotides A > G > T > C is in agreement with previous reports (37). However, the current and previously reported parameter values differ in a systematic manner, possibly reflecting the different thermal inertia of 96-well plates versus quartz cuvettes. The results for the modified DNA nucleotides are presented in Table S.5 and Figure S.11.

dangling reside	T_m (°C) for 5 μM DNA	ΔT_m (°C)	ΔH° (kcal mol^{-1})	ΔS° (cal mol^{-1}K^{-1})	ΔG°_{37} (kcal mol^{-1})	$\Delta\Delta G^\circ_{37}$ (kcal mol^{-1})
core	49.5 ± 0.4	—	37 ± 2	89 ± 6	8.9 ± 0.1	—
A	56.6 ± 0.3	7.1 ± 0.5	41 ± 3	101 ± 10	10.0 ± 0.2	1.1 ± 0.2
C	52.5 ± 0.3	3.0 ± 0.5	34 ± 1	81 ± 3	9.2 ± 0.1	0.3 ± 0.1
G	55.5 ± 0.4	6.0 ± 0.6	38 ± 4	92 ± 13	9.7 ± 0.3	0.8 ± 0.3
T	53.2 ± 0.4	3.7 ± 0.6	36 ± 2	86 ± 6	9.3 ± 0.1	0.4 ± 0.1
**2**	53.5 ± 0.2	3.9 ± 0.4	40 ± 2	97 ± 7	9.5 ± 0.1	0.6 ± 0.1
**3**	56.7 ± 0.5	7.2 ± 0.6	38 ± 4	90 ± 13	9.8 ± 0.3	0.9 ± 0.3
**4**	58.2 ± 0.6	8.7 ± 0.7	30 ± 4	67 ± 12	9.5 ± 0.3	0.6 ± 0.3
**6**	53.5 ± 0.4	4.0 ± 0.6	55 ± 5	144 ± 15	10.3 ± 0.3	1.4 ± 0.3
**9**	61.3 ± 0.6	11.8 ± 0.7	42 ± 7	101 ± 22	10.6 ± 0.6	1.7 ± 0.6

Table S.5: Free energy of stacking ($\Delta\Delta G^\circ_{37}$) for natural and modified DNA nucleotides determined from dangling end thermal denaturation.

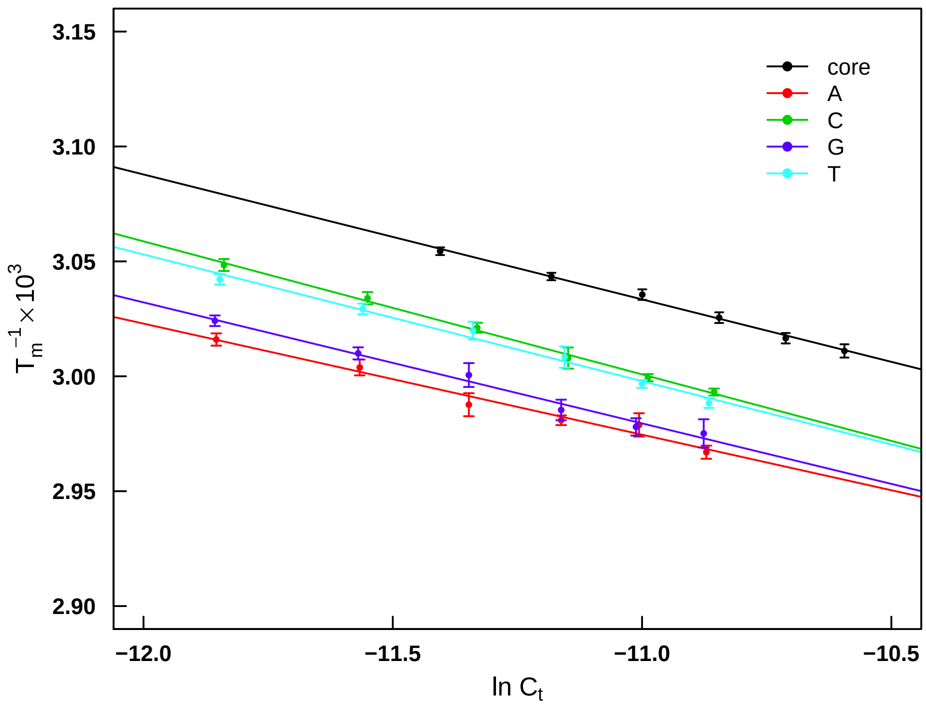

Figure S.10: van't Hoff plots of natural DNA nucleotides.

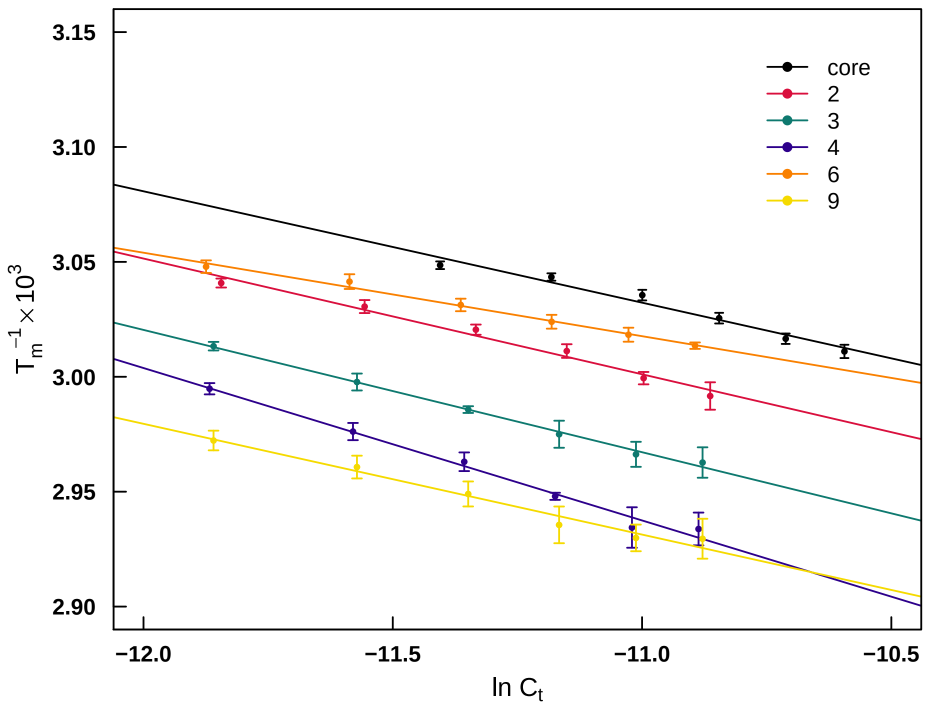

Figure S.11: van't Hoff plots of modified DNA nucleotides.

S.2.5 Thermal Denaturation of Modified DNAs

The thermal denaturation data of the 418-bp modified DNAs were also processed following the two-state transition model (27). After converting the data using Equation S.8, the equilibrium constant as a function of temperature K_a was determined (assuming a non-self-complementary bimolecular reaction)

$$K_a(T) = \frac{\theta}{D_0(1-\theta)^2} \tag{S.9}$$

where D_0 is the initial DNA concentration. The enthalpy (ΔH°) and entropy (ΔS°) parameters (with all the assumptions detailed above) were determined by fitting (linear regression analysis) with the classic van't Hoff equation (Equation S.3), the midpoint transition temperature (T_m) was evaluated from $\theta = 0.5$, and free energy (ΔG°_{37}) was calculated from the fitted values (Equation S.1). Table S.6 compares the thermal stabilities of the modified DNAs with estimates of thermodynamic parameters characterizing the melting transition.

DNA	T_m (°C)	ΔH° (kcal mol^{-1})	ΔS° (cal mol^{-1} K^{-1})	ΔG°_{37} (kcal mol^{-1})
**1**	87.1 ± 0.5	400 ± 50	1100 ± 130	56 ± 7
**2**	85.3 ± 0.6	460 ± 80	1280 ± 230	62 ± 11
**3**	93.3 ± 0.7	440 ± 30	1200 ± 90	69 ± 6
**4**	91.5 ± 0.7	400 ± 20	1100 ± 50	61 ± 2
**5**	88.3 ± 0.3	240 ± 10	670 ± 30	35 ± 2
**6**	86.5 ± 0.7	280 ± 20	780 ± 40	39 ± 2
**7**	93.4 ± 0.3	450 ± 50	1210 ± 150	70 ± 7
**8**	86.8 ± 0.4	170 ± 20	480 ± 60	24 ± 3
**9**	93.0 ± 0.5	490 ± 30	1350 ± 80	76 ± 5

Table S.6: Estimates of thermodynamic parameters from thermal denaturation of modified DNAs.

S.2.6 Ultraviolet (UV) Absorption Spectroscopy

Absorption spectra were acquired at 25°C on a double beam Cary 300 UV-Visible Spectrophotometer equipped with a Cary temperature controller (Varian) using 10 mm pathway quartz cuvettes.

dNTP	λ^1_{max} (nm)	λ^2_{max} (nm)	ϵ_{260} (M^{-1}cm^{-1})
dATP	259.5 ± 0.2		15400
dCTP	272.3 ± 0.2		7400
dGTP	252.5 ± 0.2		11500
dTTP	267.1 ± 0.2		8700
**2**	262.2 ± 0.3		10060
**3**	231.4 ± 0.1	292.1 ± 0.2	4070
**4**	240.3 ± 0.2	289.4 ± 0.3	4550
**5**	301.0 ± 0.3		12290
**6**	264.0 ± 0.1	298.2 ± 0.4	12290
**7**	232.6 ± 0.1	290.8 ± 0.2	8080
**8**	231.4 ± 0.2	289.7 ± 0.2	8080
**9**	255.0 ± 0.3	281.4 ± 0.1	7730

Table S.7: Absorption and extinction data for dNTPs

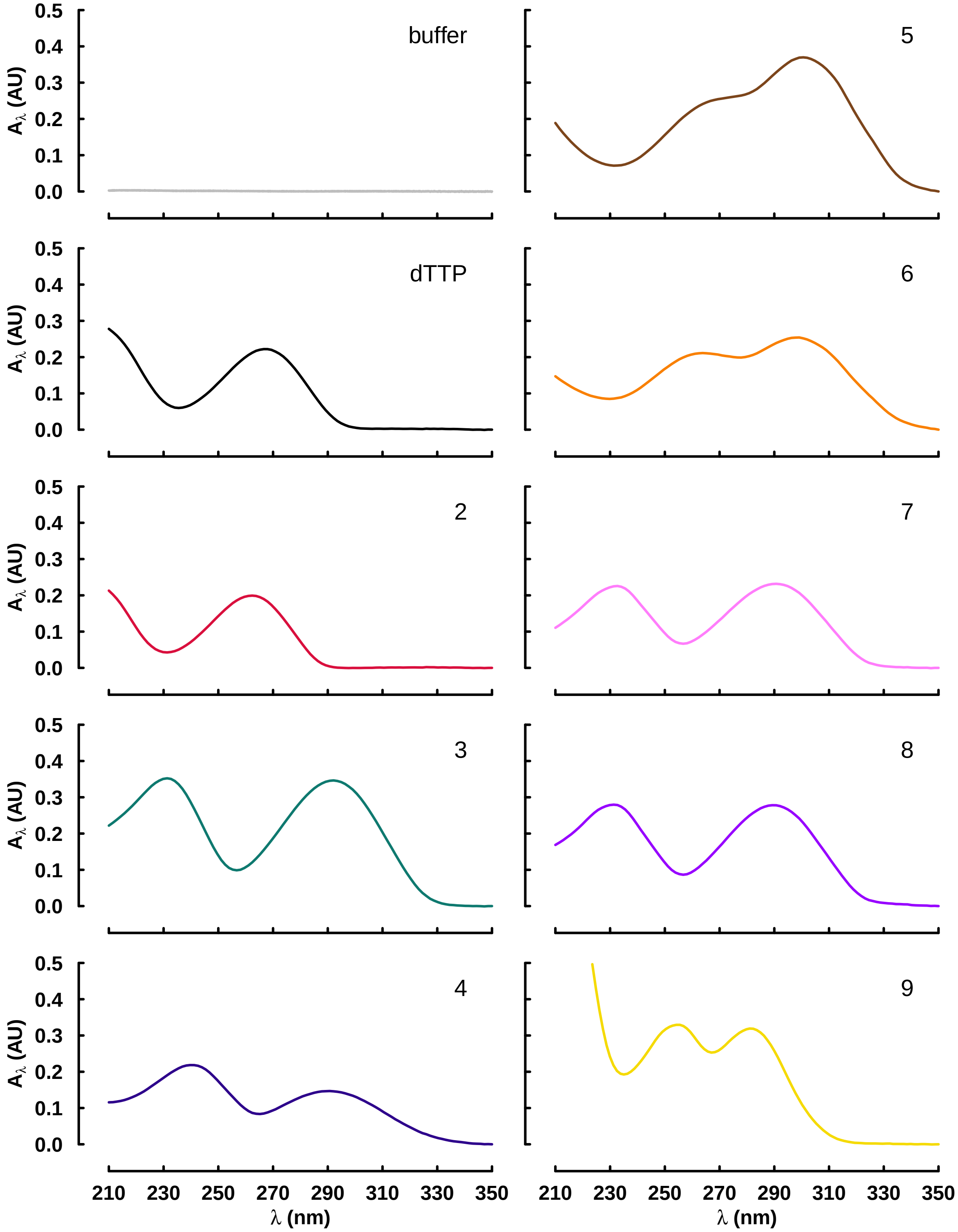

Figure S.12: Absorption spectra for the modified dNTPs

Absorption spectra were recorded in triplicate from 210 to 350 nm in 0.1 nm increments using 2.5 mM solutions of each dNTP. Figure S.12 shows the average A_λ (the absorbance at a given wavelength of light, λ) for the modified dNTPs. Table S.7 summarizes the mean and standard deviation (s.d.) for λ_{max} and highlights the differences among the spectra. When necessary, the absorption spectra allowed for conversion of the molar extinction coefficient (ϵ) from λ_{max} to the desired wavelength (260 nm), as shown in Table S.7.

S.2.7 Circular Dichroism (CD) Spectroscopy

The canonical B-DNA circular dichroism (CD) spectrum is characterized by a negative peak in the wavelength range of 245–250 nm and an approximately equal positive peak between 275–280 nm (39, 40). Thus, the CD spectrum is balanced above roughly 220 nm with the two peaks centered around 260 nm (40). It is known that the position and amplitude of CD peaks differ depending on changes in natural sequence (which alter the order of chromophores and potentially alter conformational properties), though the overall pattern is conserved (39). In general, as A/T sequence content increases, the negative peak of a B-DNA signature becomes deeper and the positive peak, which is more influenced by sequence, becomes broader (39).

For comparison, the canonical A-DNA CD spectrum has a positive peak near 270 nm and a deep negative peak around 210 nm (40). This transition from a negative peak at short wavelength to a positive peak at long wavelength indicates the bases form a right-handed stack (as is the case for B-DNA as well). However, the transition between peaks for A-DNA is characterized by a shoulder and shallow crossover (40). Finally, canonical Z-DNA has a balanced CD spectrum above 240 nm with approximately equal positive (260 nm) and negative (290 nm) peaks centered around 280 nm (40). Transition from a positive peak at short wavelength to a negative peak at long wavelength indicates the bases form a left-handed stack.

DNA	λ^- (nm)	$\lambda^{crossover}$ (nm)	λ^+ (nm)
**1**	247	261	279
**2**	246	259	273
**3**	250	257	267
**4**	251	263	269
**5**	245	251	264
**6**	244	254	263
**7**	250	256	267
**8**	246	260	274
**9**	248	275	292

Table S.8: Circular dichroism data for modified DNAs.

For the DNA variants examined here (main text Figure 3 and Table S.8), four groups emerge from the CD analysis. The first group includes natural DNA **1** as well as variants **2** and **8**. This group is the most closely aligned with canonical B-DNA, with roughly equal negative peaks occurring between 246–247 nm, positive peaks between 273–279 nm, and crossovers between 259–261 nm. The second group includes variants **3**, **4**, and **7** whose negative peaks are shifted to longer wavelengths occurring between 250–251 nm, positive peaks are shifted to shorter wavelengths between 267–269 nm, and crossovers are broadened to the range 256–263 nm. Interestingly, the positive peaks in the CD spectra of variants **3** and **7** are unchanged in intensity while the negative peaks are shallower. In contrast, the CD spectrum of variant **4** is asymmetric in the opposite

manner, with a shallower positive peak and a negative peak whose depth is unchanged though the peak appears to extend a shoulder well into shorter wavelengths. This second group also displays a low intensity negative peak near 295 nm. A negative peak around 295 nm is a pronounced, high intensity peak for the third group, variants **5** and **6**, with the depth of this peak roughly matching that of the positive peaks between 263–264 nm, reminiscent of a Z-DNA signature despite the fact the wavelengths are shifted. However, this group also contains shallow negative peaks between 244–245 nm and crossovers between 251–254 nm. These features, though shifted to shorter wavelengths, are more in line with a B-type signature. Finally, adenine substitution with diaminopurine (variant **9**) has the most noticeably distinct CD spectrum. This variant (the only member of the fourth group) exhibits a positive peak shift to 292 nm and a deep negative peak at 248 nm with a shoulder and shallow crossover near 275 nm, suggesting partial A-type character. In addition, this variant has a high intensity, broad positive peak near 225 nm not seen to the same extent in any other CD spectrum.

S.3 Cyclization Kinetics

The theoretical development of cyclization kinetics for schemes with growing complexity of reaction mechanism is sketched below, starting with a brief introduction to the cyclization kinetics approach for the simplest reaction mechanism.

S.3.1 Reaction Mechanism

The simplest reaction mechanism for cyclization is the following

$$\begin{aligned} M &\overset{k_C}{\rightarrow} C \\ M + M &\overset{k_D}{\rightarrow} D \end{aligned}$$

where the reacting species (termed linear monomer) for both unimolecular circularization and biomolecular end-joining reactions is M. Linear monomer cyclizes to form monomeric circle C at the rate k_C and undergoes a biomolecular reaction to form linear dimer D at the rate k_D. If initially the only starting material is linear monomer, then the following conservation law exists

$$[M(0)] = [M(t)] + [C(t)] + 2[D(t)] \tag{S.10}$$

For simplicity, the notation for concentration will be omitted in the following discussion and all concentrations are implicitly functions of time t. Thus rewritten

$$M_0 = M + C + 2D \tag{S.11}$$

where M_0 is the starting concentration of linear monomer. The rate of change of each species with time is given from the principle of mass action by

$$\frac{dM}{dt} = -k_C M - 4k_D M^2 \tag{S.12}$$

$$\frac{dC}{dt} = k_C M \tag{S.13}$$

$$\frac{dD}{dt} = \left(\frac{1}{2}\right) 4k_D M^2 \tag{S.14}$$

Note that the rate k_D is modulated by a statistical factor of four to account for the inability to distinguish between identical DNA termini during the dimerization of linear monomer. A statistical consideration is also necessary in Equation S.14. In this case, the number of pairs of interactions between linear monomers (as compared to interactions between two distinguishable molecules participating in a bimolecular reaction) necessitates a statistical factor of one half.

The cyclization reaction mechanism has now been expressed as a system of ordinary differential equations (ODEs). For this simple model, an analytical solution exists (55). By rewriting the conservation law (Equation S.11) as

$$D = \frac{1}{2}(M_0 - C - M) \tag{S.15}$$

the solution takes the form

$$\begin{aligned} M(t) &= \frac{M_0 e^{-k_C t}}{1 + 4M_0\left(1 - e^{-k_C t}\right)\left(\frac{k_D}{k_C}\right)} && \text{(S.16)} \\ C(t) &= \frac{k_C}{4k_D}\left(-k_C t + \ln\left(\frac{M_0}{M(t)}\right)\right) && \text{(S.17)} \\ D(t) &= \frac{1}{2}\big(M_0 - C(t) - M(t)\big) && \text{(S.18)} \end{aligned}$$

This solution is used to calculate the J-factor using a curve fitting approach (shown below).

S.3.2 J-factor Estimation

Recall (1) that under the appropriate reaction conditions the J-factor (j) is related to the rates of intramolecular (k_C) and intermolecular (k_D) ligation by

$$j = \frac{k_C}{k_D} \tag{S.19}$$

Therefore, determining j means determining the rates k_C and k_D. To this end, two methods are briefly presented. The first method is the classic approach (presented for validation purposes), whereas the second method is the approach advocated in this work.

Extrapolation Method

Returning to the conservation law (Equation S.11), it follows that

$$M = M_0 - C - 2D \tag{S.20}$$

Early in the reaction ($t \approx 0$), when only a small fraction of linear monomer has been converted to either circle or dimer, it is reasonable to use the approximation $M \approx M_0$. Under these conditions

$$\begin{aligned} \frac{dC}{dt} &= k_C M &&\approx k_C M_0 && \text{(S.21)} \\ \frac{dD}{dt} &= 2k_D M^2 &&\approx 2k_D {M_0}^2 && \text{(S.22)} \end{aligned}$$

so that

$$\begin{aligned} C(t) &= k_C M_0 t && \text{(S.23)} \\ D(t) &= 2k_D {M_0}^2 t && \text{(S.24)} \end{aligned}$$

and the desired J-factor is

$$j = \frac{k_C}{k_D} = \left.\frac{\left(\frac{C(t)}{M_0 t}\right)}{\left(\frac{D(t)}{2{M_0}^2 t}\right)}\right|_{t\approx 0} = 2M_0 \left.\frac{C(t)}{D(t)}\right|_{t\approx 0} = 2M_0 \lim_{t\to 0} \frac{C(t)}{D(t)} \tag{S.25}$$

This result will be referred to as the extrapolation method. Note that this approach has the disadvantage that limited extent of reaction progress is required. Experimentally, this requirement can be difficult to assess. In contrast, fitting methods are not subject to the same constraint.

Fitting Method

Using Equation S.16, Equation S.17, and Equation S.18 as the model $\mathbf{X}(t;\boldsymbol{\theta})$

$$\mathbf{X}(t;\boldsymbol{\theta}) = \begin{bmatrix} M(t; M_0, k_C, k_D) \\ C(t; M_0, k_C, k_D) \\ D(t; M_0, k_C, k_D) \end{bmatrix} \tag{S.26}$$

a weighted nonlinear least squares method can be used to minimize the cost function $C(\boldsymbol{\theta})$ with respect to the free parameters $\boldsymbol{\theta}$

$$\chi^2 = \min_{\boldsymbol{\theta}} C(\boldsymbol{\theta}) = \min_{\boldsymbol{\theta}} \sum_{n=1}^{N} \left(\frac{\bar{\mathbf{X}}_n - \mathbf{X}(t_n;\boldsymbol{\theta})}{\boldsymbol{\sigma}_n} \right)^2 \tag{S.27}$$

where N is the number of data points being fit (for each species), and (corresponding to the n-th data point) $\bar{\mathbf{X}}_n$ are experimentally determined mean concentrations, $\boldsymbol{\sigma}_n$ are standard deviations of $\bar{\mathbf{X}}_n$ (or another choice of weights), and $\mathbf{X}(t_n;\boldsymbol{\theta})$ are theoretical predictions of the model.

Note that M_0 can be included as a fitted parameter or set to a fixed value. Also, using the substitution $k_D = k_C/j$, the J-factor can be directly obtained from the fitting routine. This proves useful when estimating uncertainty since it eliminates the propagation of error required from computing a ratio. Furthermore, while the approach presented above assumes that experiments provide mean concentrations (and standard deviations) of the various species, the method can also be applied to individual experiments, in which case the experimental values are taken as means and the weights $\boldsymbol{\sigma}_n$ are fixed at one. In subsection S.3.4, fittings will be performed using both strategies. Even though both of these strategies involve the fitting of data from several different species, these fits are considered "individual fits" since the entire data set arises from a single experiment (or mean-value data are generated from repeats of the same experiment). In contrast "global fits" involve fitting data sets from multiple distinct experiments. If there are K separate experiments, then for the k-th experiment a cost function C_k can be defined which depends on a subset of the parameters $\boldsymbol{\theta}_k$ and the global chi-square is given by

$$\chi_G^2 = \min_{\boldsymbol{\theta}} \sum_{k=1}^{K} C_k(\boldsymbol{\theta}_k) \tag{S.28}$$

The necessity or not of global fitting will be discussed in subsection S.3.5.

S.3.3 Increasing Mechanism Complexity

Moving beyond the simple reaction mechanism, it is evident that linear species (if produced in enough abundance) can continue to cyclize or multimerize. Accordingly, the next simplest reaction mechanism and ODE system are given in Figure S.13. This model does not have an analytical solution. However, numerical methods are up to the task. Thus, the fitting method (as presented in subsection S.3.2) can be employed with one addition step, i.e. using a numerical ODE solver to generate the model $\mathbf{X}(t;\boldsymbol{\theta})$ (concentration curves as a function of time).

From here, the reaction mechanism can be expanded by adding two additional species at each increment (a linear and circular pair). For convenience, the various reaction mechanisms will be referenced by the number of resulting equations in the ODE system. An ODE system with n equations is designated model$_n$. Thus, Figure S.13 shows model$_4$. By designating linear and circular

(a)

$$M + M \xrightarrow{k_D} D \qquad M \xrightarrow{k_{C1}} C_M$$
$$M + D \xrightarrow{k_D} \ldots \qquad D \xrightarrow{k_{C2}} C_D$$

(b)

$$\frac{d[M]}{dt} = -4k_D[M]^2 - 4k_D[M][D] - k_{C1}[M]$$
$$\frac{d[D]}{dt} = 2k_D[M]^2 - 4k_D[D][M] - 4k_D[D]^2 - k_{C2}[D]$$
$$\frac{d[C_M]}{dt} = k_{C1}[M]$$
$$\frac{d[C_D]}{dt} = k_{C2}[D]$$

Figure S.13: Scheme for DNA cyclization kinetics with four distinct species. (a) Left: Intermolecular ligation reactions giving rise to linear products. The symbol "…" indicates that linear trimer does not appreciable accumulate to be detected. Right, intramolecular cyclization reactions giving rise to cyclic (C_x) products, where x refers to linear monomer M or linear dimer D. Rate constants are indicated and assumed equal for all intermolecular ligations. (b) ODE system describing accumulation of all species up to (linear and circular) dimer.

species L_i and C_i, respectively, the arbitrary model system (model$_n$) can be written succinctly as

$$\frac{dL_i}{dt} = 2k_D\left(\sum_{k=1}^{i-1} L_k L_{i-k}\right) - 4k_D L_i\left(\sum_{k=1}^{n} L_k\right) - k_{Ci}L_i \tag{S.29}$$

$$\frac{dC_i}{dt} = k_{Ci}L_i \tag{S.30}$$

with initial conditions $[L_1](0) = M_0$, $[L_i](0) = 0$ for $i > 1$, and $[C_i](0) = 0$ for all i. Fitted values for the rates of intramolecular (k_{Ci}) and intermolecular (k_D) ligations are used to determine the desired J-factors

$$j_i = \frac{k_{Ci}}{k_D} \tag{S.31}$$

where $i = 1$ for monomer cyclization.

S.3.4 Assessing the Robustness of the Fitting Approach

The starting point for this analysis is shown in Figure S.14. This figure compares the extrapolation and fitting methods. Both methods give comparable results for the J-factor, $j = 0.9$ for the extrapolation method and $j = 1.0$ for the fitting method. Decreasing the frequency of time points did not affect this result. In particular, for this probe with four time points $j = 1.0 \pm 0.1$ (below).

To assess the quality of the curve fitting approach of the various models in a comprehensive manner, all of the data from one modified DNA (**5**) are now analyzed. This includes 4 replicate experiments at each of 6 lengths. In the figures that follow, the data will be presented as an individual experiment (with detailed axes and figure legends) followed by the complete panel of the 24 experiments (using the same axes and legends).

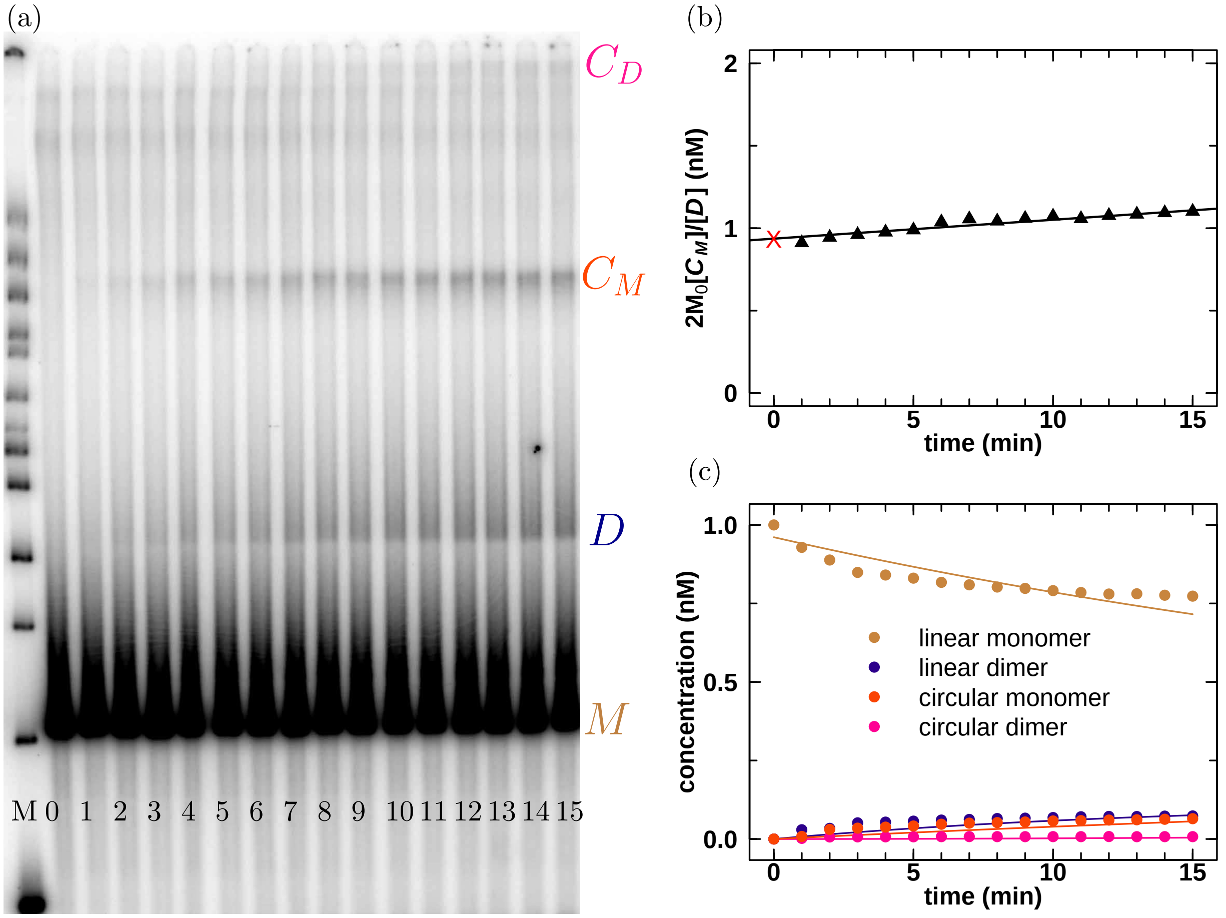

Figure S.14: Comparison of two methods of cyclization kinetics data analysis. (a) Imaged native polyacrylamide gel. Lanes contains 100 bp ladder (M), linear monomer without ligase (0), and increasing 1 min time points of the ligation reaction (1–15) showing the evolution of linear monomer (M), linear dimer (D), circular monomer (C_M) and circular dimer (C_D). (b) Extrapolation method based on the ratio of monomer circle to linear dimer ($[C_M]/[D]$). The J-factor estimate is given by twice the initial DNA concentration (M_0) multiplied by the ratio extrapolated to time zero (red cross). (c) Fitting method based on cyclization kinetics with model$_4$.

Extrapolation versus Curve Fitting

First, J-factors are determined using the extrapolation method, Figure S.15. If the first data point (5 min) appeared anomalous, only the 10, 15, and 20 min time points were used for the linear regression. Each experiment is also independently fit with each of model$_4$, model$_6$, model$_8$, and model$_{10}$, as shown in Figure S.16. Table S.9 summarizes the differences between the extrapolation and fitting (model$_4$, model$_6$, model$_8$, and model$_{10}$) methods. It should be noted that the values of the parameters determined (using model$_4$) by fitting only the the 10, 15, and 20 min time points fall within the ranges presented in Table S.9. This result suggests that the fitting method is therefore less sensitive to outliers than the extrapolation method. Also, as mentioned previously, this confirms that only a limited number of data points are required for reliable parameter determination. Finally, only the fitting approach allows for determination of the rates k_C and k_D (Table S.9). These rates can be utilized in gauging quality or in constraining global fits (see subsection S.3.5).

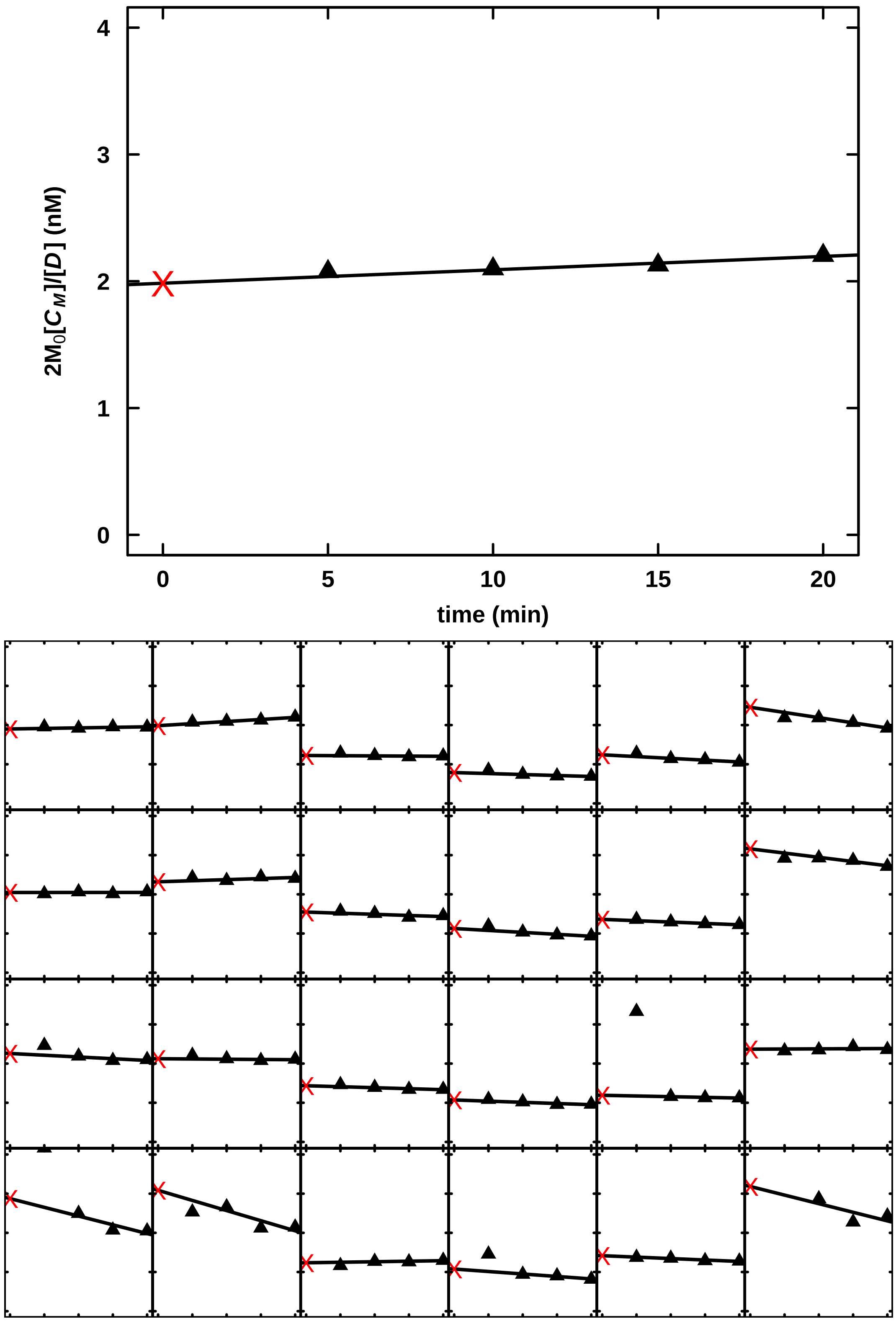

Figure S.15: Extrapolation method.

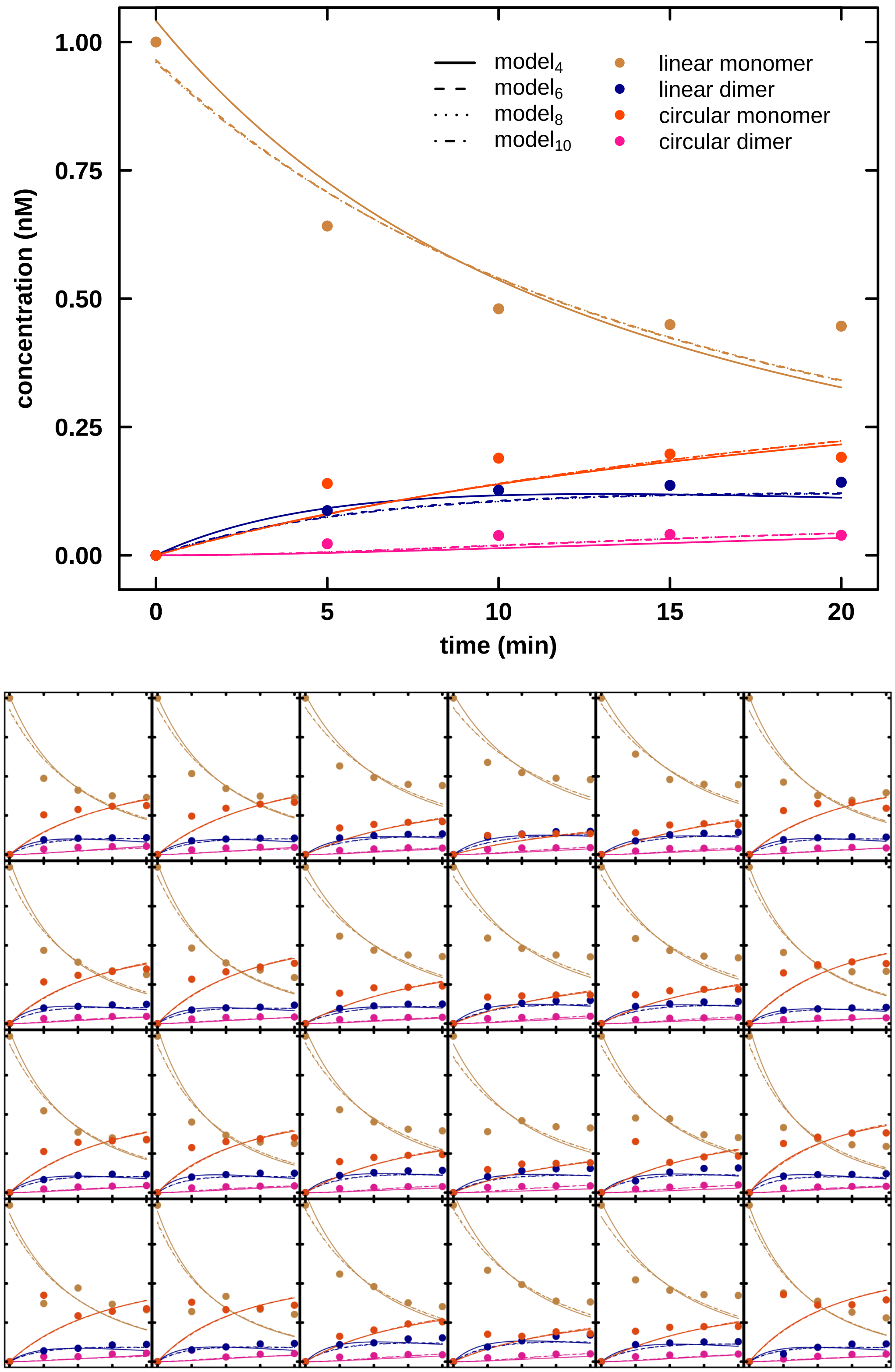

Figure S.16: Fitting method.

DNA length (bp)	extrapolation	$model_4$	$model_6$	$model_8$	$model_{10}$
	J-factor ± s.d. (nM)				
201	2.3 ± 0.4	2.3 ± 0.3	2.8 ± 0.2	2.9 ± 0.2	2.9 ± 0.2
203	2.4 ± 0.5	2.4 ± 0.2	2.9 ± 0.2	2.9 ± 0.2	3.0 ± 0.2
205	1.4 ± 0.2	1.5 ± 0.1	1.8 ± 0.2	1.9 ± 0.1	1.9 ± 0.1
207	1.0 ± 0.2	1.0 ± 0.1	1.2 ± 0.2	1.3 ± 0.2	1.3 ± 0.2
209	1.3 ± 0.1	1.4 ± 0.1	1.7 ± 0.1	1.7 ± 0.1	1.7 ± 0.1
211	2.8 ± 0.4	2.6 ± 0.5	3.2 ± 0.5	3.3 ± 0.5	3.3 ± 0.5
		k_{C1} ± s.d. (× 10^{-2} s^{-1})			
201		4.0 ± 0.3	4.1 ± 0.3	4.2 ± 0.3	4.2 ± 0.3
203		4.4 ± 0.5	4.6 ± 0.5	4.6 ± 0.5	4.6 ± 0.5
205		2.4 ± 0.2	2.5 ± 0.2	2.5 ± 0.2	2.5 ± 0.2
207		1.7 ± 0.4	1.8 ± 0.4	1.8 ± 0.4	1.8 ± 0.4
209		2.3 ± 0.4	2.4 ± 0.4	2.4 ± 0.4	2.4 ± 0.4
211		4.9 ± 0.6	5.0 ± 0.6	5.0 ± 0.7	5.0 ± 0.7
		k_D ± s.d. (× 10^{-2} $nM^{-1}s^{-1}$)			
201		1.8 ± 0.2	1.5 ± 0.1	1.5 ± 0.1	1.5 ± 0.1
203		1.9 ± 0.2	1.6 ± 0.2	1.6 ± 0.2	1.5 ± 0.2
205		1.6 ± 0.2	1.4 ± 0.1	1.3 ± 0.1	1.3 ± 0.1
207		1.7 ± 0.2	1.5 ± 0.2	1.4 ± 0.1	1.4 ± 0.1
209		1.6 ± 0.2	1.4 ± 0.1	1.4 ± 0.1	1.4 ± 0.1
211		1.9 ± 0.3	1.6 ± 0.2	1.5 ± 0.2	1.5 ± 0.2

Table S.9: Comparison of extrapolation and fitting ($model_4$, $model_6$, $model_8$, and $model_{10}$) methods

If $model_4$ is chosen as a reference, the influence of the various methods and models on the J-factor is shown in Table S.10. While the extrapolation method gives results in good agreement with $model_4$, the additional (in this case, meaningless) parameters in $model_6$, $model_8$, and $model_{10}$ are increasingly able to pull the value of the J-factor away from its true value. In particular, k_D appears to be more influenced by the inclusion of additional free parameters (Table S.9). Thus, using the minimal model necessary for the data is favored.

DNA length (bp)	percent of $model_4$ J-factor				
	extrapolation	$model_4$	$model_6$	$model_8$	$model_{10}$
201	99	100	122	125	125
203	100	100	123	125	125
205	93	100	124	126	126
207	103	100	124	127	128
209	94	100	123	125	125
211	108	100	123	126	126

Table S.10: Comparison of J-factors from extrapolation and fitting methods

Weighted Fitting

Next, the influence of the choice of weight on the J-factor is examined. The average value of the four replicate data sets (for each length) was determined along with the standard deviation (s.d.).

Table S.11 shows a comparison of weighted fits using model_4 and either a constant weight or weights equal to s.d.

length (bp)	weights = 1	weights = s.d.
	J-factor (nM)	
201	2.3	2.2
203	2.4	2.3
205	1.5	1.4
207	1.0	1.0
209	1.4	1.3
211	2.6	2.4
	k_{C1} ($\times\ 10^{-2}\ \text{s}^{-1}$)	
201	4.0	3.3
203	4.4	3.8
205	2.4	2.4
207	1.7	1.7
209	2.3	2.3
211	4.8	4.4
	k_D ($\times\ 10^{-2}\ \text{nM}^{-1}\text{s}^{-1}$)	
201	1.8	1.5
203	1.9	1.6
205	1.6	1.7
207	1.7	1.8
209	1.6	1.8
211	1.9	1.8

Table S.11: Dependence of fit parameters on weights

As expected, fitting the average data set (with weight of 1) is virtually the same as fitting each individual data set (with weight of 1) and then taking the average (c.f. Table S.9). However, with the limited number of replicates, anomalous values in one data set can greatly influence the fitted values of the parameters when weights are apportioned according to the s.d. (Table S.11). Therefore, weights are fixed at one in subsequent analysis.

S.3.5 Global Fitting

Recall (1) that k_D is assumed to depend only on the terminal segments undergoing biomolecular ligation, e.g. specific orientation and sequence of the DNA overhang resulting from restriction digest. As seen from Table S.9, this parameter does show subtle variation with DNA length from individual fits. Therefore, it is important to assess whether global fitting with this parameter fixed across multiple experimental data sets alters the fitted values of the J-factor. Using model_4, six fitting conditions were examined with parameters either allowed to vary by experiment, shared within the set of experiments for a given DNA length, or shared for all experimental data sets. These conditions are shown in Table S.12. Fit_1 is just the independent fitting of the 24 experiments already discussed. Fit_2 requires 6 fits each with 4 free parameters, fit_3 requires 6 fits each with 9 free parameters, fit_4 requires 6 fits each with 19 free parameters, fit_5 requires 1 fit with 37 free parameters, and fit_6 requires 1 fit with 73 free parameters. Plot of these fits are shown in Figure S.17 and the fitted values are given in Table S.13.

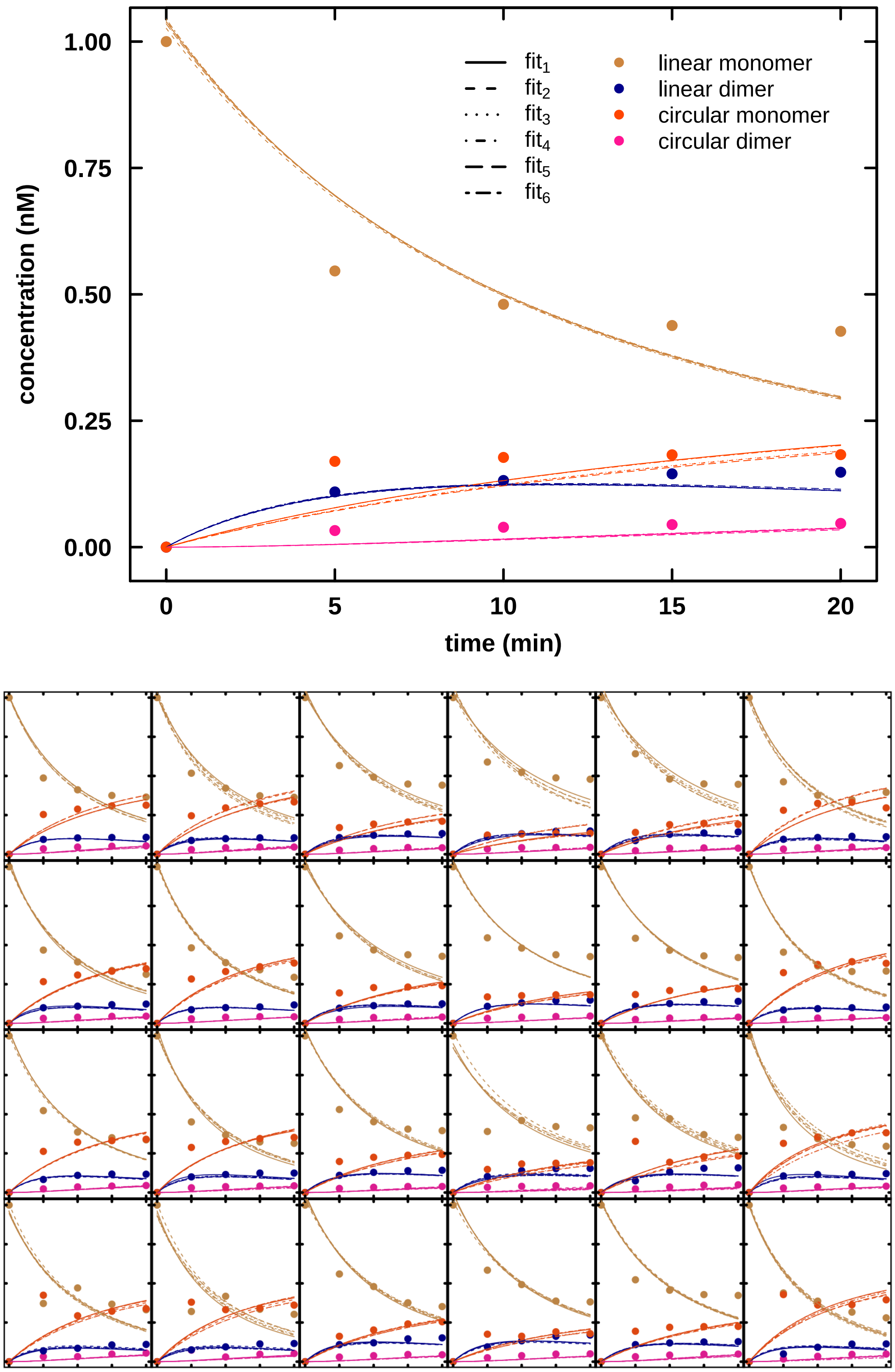

Figure S.17: Global fitting.

fitting condition	parameters		
	individual	shared by DNA length	shared by all
fit_1	k_{C1}, k_{C2}, k_D, M_0		
fit_2		k_{C1}, k_{C2}, k_D, M_0	
fit_3	M_0	k_{C1}, k_{C2}, k_D	
fit_4	k_{C1}, k_{C2}, M_0	k_D	
fit_5	M_0	k_{C1}, k_{C2}	k_D
fit_6	k_{C1}, k_{C2}, M_0		k_D

Table S.12: Global fitting conditions

length (bp)	fit_1	fit_2	fit_3	fit_4	fit_5	fit_6
	J-factor ± s.d. (nM)					
201	2.3 ± 0.3	2.3	2.3	2.3 ± 0.2	2.3	2.3 ± 0.2
203	2.4 ± 0.2	2.4	2.4	2.4 ± 0.3	2.5	2.5 ± 0.3
205	1.5 ± 0.1	1.5	1.5	1.5 ± 0.2	1.4	1.4 ± 0.1
207	1.0 ± 0.1	1.0	1.0	1.0 ± 0.2	1.0	1.0 ± 0.2
209	1.4 ± 0.1	1.4	1.4	1.4 ± 0.2	1.3	1.3 ± 0.2
211	2.6 ± 0.5	2.6	2.6	2.6 ± 0.3	2.8	2.6 ± 0.4
	k_{C1} ± s.d. ($\times\ 10^{-2}\ \text{s}^{-1}$)					
201	4.0 ± 0.3	4.0	4.0	4.0 ± 0.3	4.0	4.0 ± 0.3
203	4.4 ± 0.5	4.4	4.4	4.4 ± 0.5	4.4	4.4 ± 0.5
205	2.4 ± 0.2	2.4	2.4	2.4 ± 0.2	2.4	2.4 ± 0.2
207	1.7 ± 0.4	1.7	1.7	1.7 ± 0.4	1.7	1.7 ± 0.4
209	2.3 ± 0.4	2.3	2.3	2.3 ± 0.4	2.3	2.3 ± 0.4
211	4.9 ± 0.6	4.8	4.8	4.9 ± 0.6	4.8	4.5 ± 0.6
	k_D ± s.d. ($\times\ 10^{-2}\ \text{nM}^{-1}\text{s}^{-1}$)					
201	1.8 ± 0.2	1.8	1.8	1.8	1.7	1.7
203	1.9 ± 0.2	1.9	1.9	1.8	1.7	1.7
205	1.6 ± 0.2	1.6	1.6	1.6	1.7	1.7
207	1.7 ± 0.2	1.7	1.7	1.7	1.7	1.7
209	1.6 ± 0.2	1.6	1.6	1.6	1.7	1.7
211	1.9 ± 0.3	1.9	1.9	1.9	1.7	1.7

Table S.13: Comparison of globally fit parameters

From Table S.13, the striking result of this analysis is that global constraint of the parameter k_D does not appreciably alter the fitted values of the J-factor. Due to the greatly increased computational burden of these global fitting conditions (particularly fit_6), the original independent fitting approach (fit_1) is favored.

S.3.6 Cyclization Control Experiments

Figure S.18 demonstrates the stability of interaction between various free DNA termini as a function of temperature (56). The readout of this assay is the presence or absence of stabilized interactions of linear monomer with itself (forming a smear to circular monomer) or another linear monomer (forming a smear to linear dimer).

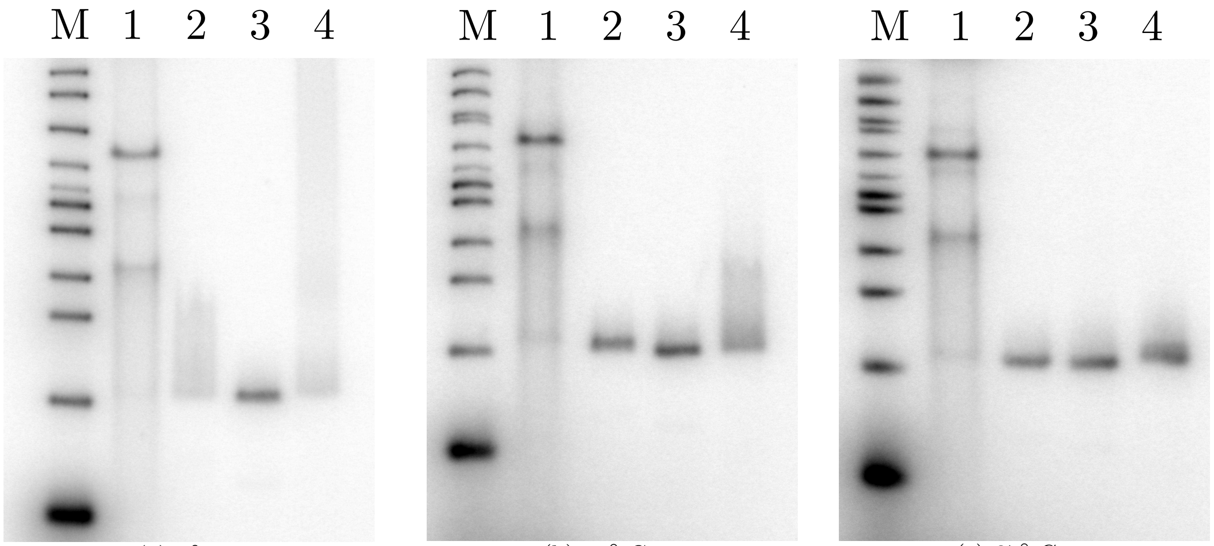

(a) 4° C (b) 25° C (c) 37° C

Figure S.18: Stabilization of free DNA termini. M indicates 100 bp ladder, and lane 1 shows the mobility of linear monomer, linear dimer, and circular monomer (from bottom to top). In lanes 2–4, experiments were performed for the 5′ single-stranded overhangs created by the restriction enzymes *Hin*dIII (5′-AGCT), *Nar*I (5′-CG), and *Ban*I (5′-GCGC), respectively. Gel electrophoresis was performed at the indicated temperatures in TBE buffer containing an additional 10 mM $MgCl_2$.

The interaction of the single-stranded termini is transient for 5′-CG ends (*Nar*I) at all temperatures examined. For 5′-AGCT overhangs (*Hin*dIII) the interaction is transient above 4° C, and for 5′-GCGC overhangs (*Ban*I) the interaction is never transient (though its prevalence decreases with temperature, as expected). Therefore, 5′-CG ends (*Nar*I) are suitable for the present cyclization experiments at room temperature.

After determining the stability of cohesive end hybridization, suitable ranges of concentrations for both DNA and DNA ligase were investigated. Each concentration was varied empirically over a wide range and J-factors determined for the 211-bp natural DNA probe. Ligase concentrations (activity units being used as a surrogate) over the range of 1 U/mL to 1000 U/mL were tested to determine when ligase begins to influence the J-factor estimates for ∼200-bp probes. The conclusion of Figure S.19 is that ligase should not exceed roughly 100 U/mL. A similar conclusion was previously reached for DNA probes as short as 100 bp (57). Next, DNA concentration was varied from 0.5 nM to 100 nM. Figure S.20 shows that 1 nM probe concentration is ideal.

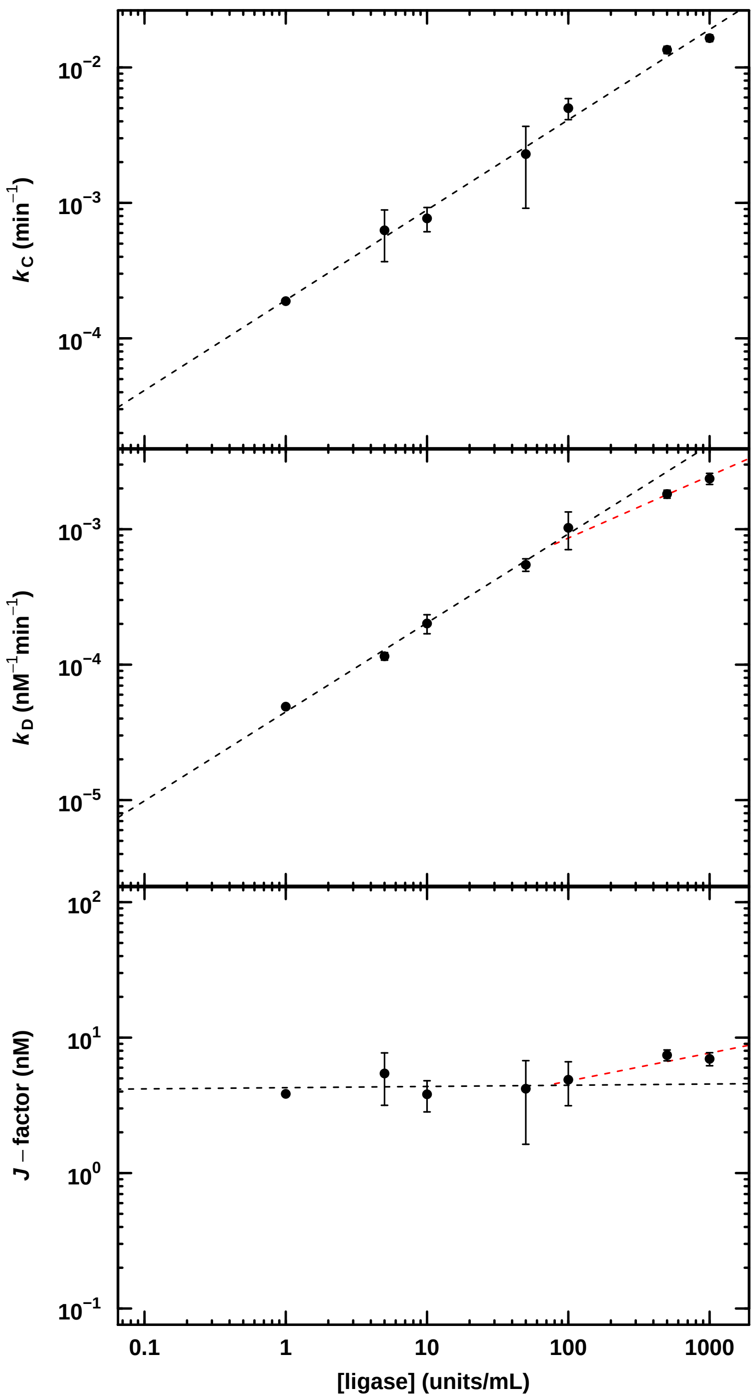

Figure S.19: Effect of ligase concentration on J-factor.

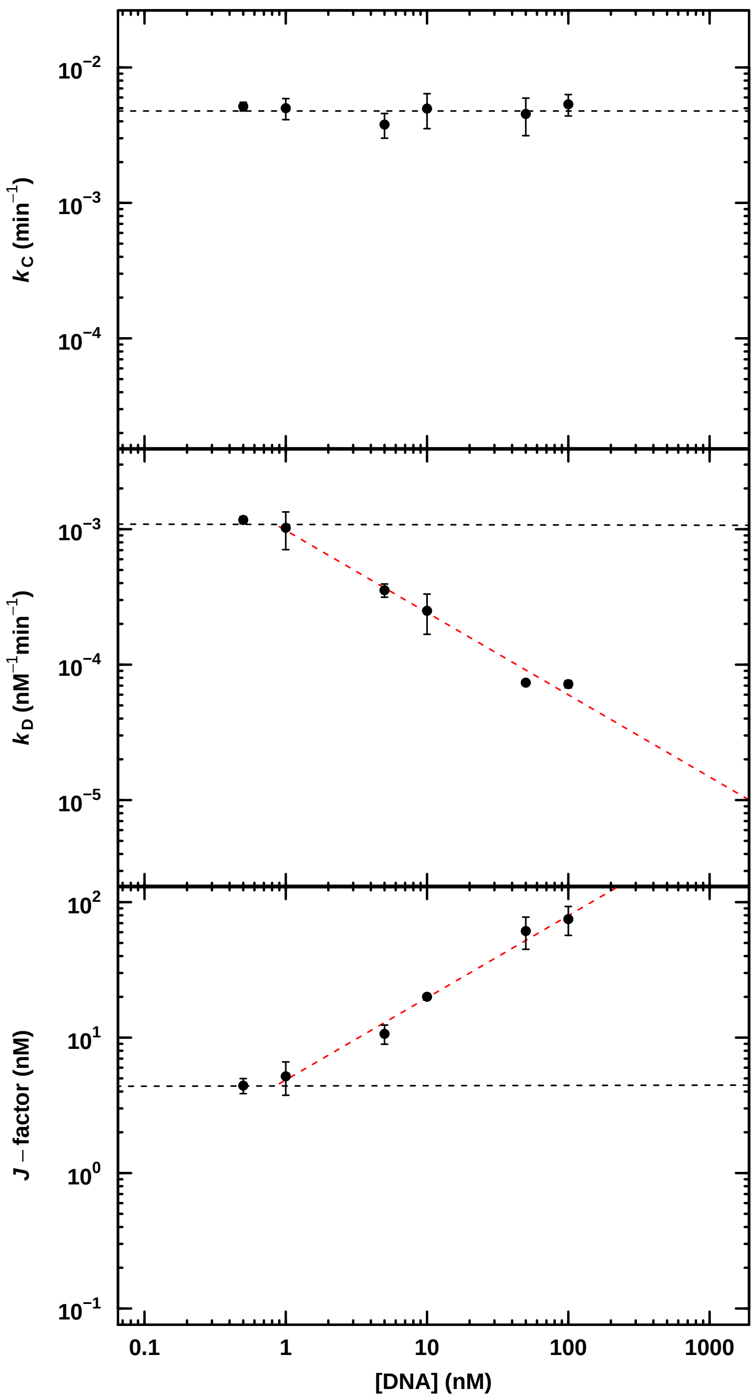

Figure S.20: Effect of DNA concentration on J-factor.

S.4 Manning Theory

S.4.1 Counterion Condensation (CC) Theory

Manning's counterion condensation (CC) theory (36) describes ion condensation on DNA as when the local concentration of cations is high and fairly independent of bulk salt. The (dimensionless) parameter ξ governs counterion binding, and is defined as follows:

$$\xi \equiv \frac{e^2}{\epsilon k T b} = \frac{\ell_B}{b} \tag{S.32}$$

where e is the fundamental charge, ϵ is the solvent dielectric constant which acts to dampen electrostatic interactions, k is the Boltzmann constant, T is the absolute temperature, and b is the average spacing between charges. The quantity $e^2/(\epsilon kT) \equiv \ell_B$ is the Bjerrum length for the pure solvent. For water at 25°C, $\ell_B = 7.14$ Å. It should be emphasized that CC only occurs when $\xi > 1$ ($b < 7.14$ Å). If $b \geq 7.14$ Å, then no ion condensation occurs.

The residual charge r remaining on the polymer after condensation of the counterions is given by:

$$r = \frac{1}{Z\xi} \tag{S.33}$$

where Z is the valence of the counterion.

For DNA-like polymers in aqueous monovalent salt (e.g. NaCl),

$$\xi = \frac{7.14 \text{ Å}}{b} \tag{S.34}$$

where b is the average spacing along the DNA (in Å) per charge. The charge fraction after CC is

$$r = \frac{1}{\xi} \tag{S.35}$$

so that the net charge Q_{net} is given by

$$Q_{\text{net}} = \frac{Q}{\xi} \tag{S.36}$$

where $Q = Nq$ is the bare charge of a DNA molecule with N charges of magnitude q.

The prediction of CC theory boils down to a very simple point. Consider a normal 100-bp DNA with axial rise of about 3.4 Å per bp (2 negative charges)

$$b = \frac{3.4 \text{ Å}}{2} = 1.7 \text{ Å} \tag{S.37}$$

so that $\xi = 4.2$ and the residual charge fraction is ~ 0.24. Since $Q = -200$,

$$Q_{\text{net}} = -\frac{200}{4.2} \approx -48 \tag{S.38}$$

Note that the total length of the polymer is given by $|Q|b = 340$ Å. Next, consider a 100-bp DNA-like polymer (of the same overall length) with 25% extra negative charge, i.e. $Q = -250$. To maintain the same total length, the average charge spacing of the polymer must decrease

$$b = \frac{340 \text{ Å}}{250} = 1.36 \text{ Å} \tag{S.39}$$

and in response the theory says that $\xi = 5.25$ and the residual charge fraction is ~ 0.19, so that the net charge of the polymer is

$$Q_{\text{net}} = -\frac{250}{5.25} \approx -48 \tag{S.40}$$

Thus, the claim is that net charges are invariant for DNA-like polymers (of a fixed length) even when their bare charges vary.

S.4.2 Manning Theory of Persistence Length

Manning recently proposed (11) a quantitative expression for the dependence of the persistence length on charge spacing:

$$P = \left(\frac{\pi}{2}\right)^{2/3} R^{4/3}(P^*)^{2/3} Z^{-2} \ell_B^{-1} \left[(2Z\xi - 1)\frac{\kappa b e^{-\kappa b}}{1 - e^{-\kappa b}} - 1 - \ln(1 - e^{-\kappa b})\right] \tag{S.41}$$

where R is the polymer radius, P^* is the persistence length of a hypothetical DNA that bears no ionic charge on its phosphodiesters, and κ is the Debye parameter (inverse of the Debye length from Debye-Hückel theory). By assuming that the only parameter changing among the different DNA-like polymers is the average bare charge spacing b, this equation predicts the resulting effects on bending persistence length, P_{Manning} of Table 1 main text.

What would the theory predict if applied using the charge spacing after counterion condensation b_{CC} instead of the bare charge spacing b? After CC, the charge spacing is given by

$$b_{\text{CC}} = \frac{L}{|Q_{\text{net}}|} \tag{S.42}$$

where L is the total length of the DNA. From the example above (subsection S.4.1), it follows that

$$b_{\text{CC}} = \frac{340 \text{ Å}}{48} = 7.14 \text{ Å} = \ell_B \tag{S.43}$$

Recall that Manning's CC theory predicts that the charge density of a DNA polymer, as long as it exceeds a certain limit, will cause compensating counterion condensation such that the predicted net thermodynamic charge is independent of bare charge density. This means that for DNA-like polymers of a fixed length, all will have the same effective charge spacing after CC, i.e. the Bjerrum length. With the (assumed) only variable now fixed, it follows that the prediction is that all of the polymers would have the same bending persistence length (at least to the level of course-graining of the theory which does not account for structural details at the base pair level).

References

1. Peters, J. P. and Maher III, L. J. (2010) DNA curvature and flexibility *in vitro* and *in vivo*. *Q. Rev. Biophys.,* **43**(1), 23–63.

2. Garcia, H. G., Grayson, P., Han, L., Inamdar, M., Kondev, J., Nelson, P. C., Phillips, R., Widom, J., and Wiggins, P. A. (2007) Biological consequences of tightly bent DNA: The other life of a macromolecular celebrity. *Biopolymers,* **85**, 115–130.

3. Ortiz, V. and de Pablo, J. (2011) Molecular origins of DNA flexibility: sequence effects on conformational and mechanical properties. *Phys. Rev. Lett.,* **106**(23), 238107.

4. Chen, H. H., Rau, D. C., and Charney, E. (1985) The flexibility of alternating dA-dT sequences. *J. Biomol. Struct. Dynam.,* **2**(4), 709–719.

5. Roychoudhury, M., Sitlani, A., Lapham, J., and Crothers, D. M. (2000) Global structure and mechanical properties of a 10-bp nucleosome positioning motif. *Proc. Natl. Acad. Sci. U. S. A.,* **97**(25), 13608–13613.

6. Fujimoto, B. S. and Schurr, J. M. (1990) Dependence of the torsional rigidity of DNA on base composition. *Nature,* **344**(6262), 175–177.

7. Geggier, S. and Vologodskii, A. V. (2010) Sequence dependence of DNA bending rigidity. *Proc. Natl. Acad. Sci. U. S. A.,* **107**(35), 15421–15426.

8. Lankaš, F., Šponer, J., Hobza, P., and Langowski, J. (2000) Sequence-dependent elastic properties of DNA. *J. Mol. Biol.,* **299**(3), 695–709.

9. Olson, W. K., Gorin, A. A., Lu, X.-J., Hock, L. M., and Zhurkin, V. B. (1998) DNA sequence-dependent deformability deduced from protein–DNA crystal complexes. *Proc. Natl. Acad. Sci. U. S. A.,* **95**(19), 11163–11168.

10. Yakovchuk, P., Protozanova, E., and Frank-Kamenetskii, M. (2006) Base-stacking and base-pairing contributions into thermal stability of the DNA double helix. *Nucleic Acids Res.,* **34**(2), 564–574.

11. Manning, G. S. (2006) The persistence length of DNA is reached from the persistence length of its null isomer through an internal electrostatic stretching force. *Biophys. J.,* **91**(10), 3607–3616.

12. Skolnick, J. and Fixman, M. (1977) Electrostatic persistence length of a wormlike polyelectrolyte. *Macromolecules,* **10**(5), 944–948.

13. Odijk, T. (1977) Polyelectrolytes near the rod limit. *J. Polym. Sci. B,* **15**(3), 477–483.

14. Cherstvy, A. G. (2011) DNA cyclization: suppression or enhancement by electrostatic repulsions?. *J. Phys. Chem. B,* **115**(15), 4286–4294.

15. Savelyev, A., Materese, C. K., and Papoian, G. A. (2011) Is DNA's rigidity dominated by electrostatic or nonelectrostatic interactions?. *J. Am. Chem. Soc.,* **133**(48), 19290–19293.

16. Guo, Z., Taubes, C. H., Oh, J.-E., Maher III, L. J., and Mohanty, U. (2008) DNA on a tube: electrostatic contribution to stiffness. *J. Phys. Chem. B,* **112**(50), 16163–16169.

17. McFail-Isom, L., Sines, C. C., and Williams, L. D. (1999) DNA structure: cations in charge?. *Curr. Opin. Struct. Biol.,* **9**(3), 298–304.

18. Moulaei, T., Maehigashi, T., Lountos, G. T., Komeda, S., Watkins, D., Stone, M. P., Marky, L. A., Li, J.-S., Gold, B., and Williams, L. D. (2005) Structure of B-DNA with cations tethered in the major groove. *Biochemistry,* **44**(20), 7458–7468.

19. Hud, N. V. and Polak, M. (2001) DNA-cation interactions: The major and minor grooves are flexible ionophores. *Curr. Opin. Struct. Biol.,* **11**(3), 293–301.

20. Mills, J. B. and Hagerman, P. J. (2004) Origin of the intrinsic rigidity of DNA. *Nucleic Acids Res.,* **32**(13), 4055–4059.

21. Kosikov, K. M., Gorin, A. A., Lu, X.-J., Olson, W. K., and Manning, G. S. (2002) Bending of DNA by asymmetric charge neutralization: all-atom energy simulations. *J. Am. Chem. Soc.,* **124**(17), 4838–4847.

22. Manning, G. S. (2003) Is a small number of charge neutralizations sufficient to bend nucleosome core DNA onto its superhelical ramp?. *J. Am. Chem. Soc.,* **125**(49), 15087–15092.

23. Mirzabekov, A. and Rich, A. (1979) Asymmetric lateral distribution of unshielded phosphate groups in nucleosomal DNA and its role in DNA bending. *Proc. Natl. Acad. Sci. U. S. A.,* **76**(3), 1118–1121.

24. Range, K., Mayaan, E., Maher III, L. J., and York, D. M. (2005) The contribution of phosphate-phosphate repulsions to the free energy of DNA bending. *Nucleic Acids Res.,* **33**(4), 1257–1268.

25. Strauss, J. K. and Maher III, L. J. (1994) DNA bending by asymmetric phosphate neutralization. *Science,* **266**(5192), 1829–1834.

26. Vologodskaia, M. Y. and Vologodskii, A. V. (2002) Contribution of the intrinsic curvature to measured DNA persistence length. *J. Mol. Biol.,* **317**(2), 205–213.

27. Mergny, J. and Lacroix, L. (2003) Analysis of thermal melting curves. *Oligonucleotides,* **13**(6), 515–537.

28. Schweitzer, B. and Kool, E. (1995) Hydrophobic, non-hydrogen-bonding bases and base pairs in DNA. *J. Am. Chem. Soc.,* **117**(7), 1863–1872.

29. Peters, J. P., Becker, N. A., Rueter, E. M., Bajzer, Ž., Kahn, J. D., and Maher III, L. J. (2011) Quantitative methods for measuring DNA flexibility *in vitro* and *in vivo*. *Methods Enzymol.,* **488**, 287–335.

30. Shimada, J. and Yamakawa, H. (1984) Ring-closure probabilities for twisted wormlike chains. Application to DNA. *Macromolecules,* **17**(4), 689–698.

31. Vologodskii, A. V., Anshelevich, V. V., Lukashin, A. V., and Frank-Kamenetskii, M. D. (1979) Statistical mechanics of supercoils and the torsional stiffness of the DNA double helix. *Nature,* **280**(5720), 294–298.

32. Klenin, K. V., Vologodskii, A. V., Anshelevich, V. V., Klishko, V. Y., Dykhne, A. M., and Frank-Kamenetskii, M. D. (1989) Variance of writhe for wormlike DNA rings with excluded volume. *J. Biomol. Struct. Dynam.,* **6**(4), 707–714.

33. Offord, C. and Bajzer, Ž. (2001) A hybrid global optimization algorithm involving simplex and inductive search. In *International conference on computational science* pp. 680–688 Springer.

34. Sakthivel, K. and Barbas III, C. (1998) Expanding the potential of DNA for binding and catalysis: highly functionalized dUTP derivatives that are substrates for thermostable DNA polymerases. *Angew. Chem. Int. Edit.,* **37**(20), 2872–2875.

35. Jäger, S., Rasched, G., Kornreich-Leshem, H., Engeser, M., Thum, O., and Famulok, M. (2005) A versatile toolbox for variable DNA functionalization at high density. *J. Am. Chem. Soc.,* **127**(43), 15071–15082.

36. Manning, G. S. (1978) The molecular theory of polyelectrolyte solutions with applications to the electrostatic properties of polynucleotides.. *Q. Rev. Biophys.,* **11**(2), 179–246.

37. Guckian, K., Schweitzer, B., Ren, R., Sheils, C., Tahmassebi, D., and Kool, E. (2000) Factors contributing to aromatic stacking in water: evaluation in the context of DNA. *J. Am. Chem. Soc.,* **122**(10), 2213–2222.

38. Virstedt, J., Berge, T., Henderson, R. M., Waring, M. J., and Travers, A. A. (2004) The influence of DNA stiffness upon nucleosome formation. *J. Struct. Biol.,* **148**(1), 66–85.

39. Kypr, J., Kejnovska, I., Renciuk, D., and Vorlickova, M. (2009) Circular dichroism and conformational polymorphism of DNA. *Nucleic Acids Res.,* **37**(6), 1713–1725.

40. Ranjbar, B. and Gill, P. (2009) Circular dichroism techniques: biomolecular and nanostructural analyses-a review. *Chem. Biol. Drug Des.,* **74**(2), 101–120.

41. Yuan, C., Lou, X. W., Rhoades, E., Chen, H., and Archer, L. A. (2007) T4 DNA ligase is more than an effective trap of cyclized dsDNA. *Nucleic Acids Res.,* **35**(16), 5294–5302.

42. Vologodskii, A. and D. Frank-Kamenetskii, M. (2013) Strong bending of the DNA double helix. *Nucleic Acids Research,*.

43. Celedon, A., Wirtz, D., and Sun, S. (2010) Torsional mechanics of DNA are regulated by small-molecule intercalation. *J. Phys. Chem. B,* **114**(50), 16929–16935.

44. Douglas, S. M., Bachelet, I., and Church, G. M. (2012) A logic-gated nanorobot for targeted transport of molecular payloads. *Sci. Signal.,* **335**(6070), 831–834.

45. Record Jr, M. T., deHaseth, P. L., and Lohman, T. M. (1977) Interpretation of monovalent and divalent cation effects on the lac repressor-operator interaction. *Biochemistry,* **16**(22), 4791–4796.

46. Record Jr, M. T., Zhang, W., and Anderson, C. F. (1998) Analysis of effects of salts and uncharged solutes on protein and nucleic acid equilibria and processes: a practical guide to recognizing and interpreting polyelectrolyte effects, Hofmeister effects, and osmotic effects of salts. *Adv. Protein Chem.,* **51**, 281–353.

47. Čapek, P., Cahová, H., Pohl, R., Hocek, M., Gloeckner, C., and Marx, A. (2007) An efficient method for the construction of functionalized DNA bearing amino acid groups through cross-coupling reactions of nucleoside triphosphates followed by primer extension or PCR. *Chem. Eur. J.,* **13**(21), 6196–6203.

48. Kovács, T. and Ötvös, L. (1988) Simple synthesis of 5-vinyl- and 5-ethynyl-2′deoxyuridine-5′-triphosphates. *Tetrahedron Lett.,* **29**(36), 4525–4528.

49. Moffatt, J. (1964) A general synthesis of nucleosides-5′triphosphates. *Can. J. Chem.,* **42**(3), 599–604.

50. Sorensen-Stowell, K. and Hengge, A. (2005) Examination of P-OR bridging bond orders in phosphate monoesters using ^{18}O isotope shifts in ^{31}P NMR. *J. Org. Chem.,* **70**(12), 4805–4809.

51. Berkner, K. L. and Folk, W. R. (1979) The effects of substituted pyrimidines in DNAs on cleavage by sequence-specific endonucleases. *J. Biol. Chem.,* **254**(7), 2551–2560.

52. Yu, L., Zhao, J., Li, S. P., Fan, H., Hong, M., Wang, Y. T., and Zhu, Q. (2006) Quality evaluation of *Cordyceps* through simultaneous determination of eleven nucleosides and bases by RP-HPLC. *J. Sep. Sci.,* **29**(7), 953–958.

53. Kuo, K. C., McCune, R. A., Gehrke, C. W., Midgett, R., and Ehrlich, M. (1980) Quantitative reversed-phase high performance liquid chromatographic determination of major and modified deoxyribonucleosides in DNA. *Nucleic Acids Res.,* **8**(20), 4763–4776.

54. Chaires, J. B. (1997) Possible origin of differences between van't Hoff and calorimetric enthalpy estimates. *Biophys. Chem.,* **64**(1), 15–23.

55. Kahn, J. D. and Crothers, D. M. (1992) Protein-induced bending and DNA cyclization. *Proc. Natl. Acad. Sci. U. S. A.,* **89**(14), 6343–6347.

56. Geggier, S., Kotlyar, A., and Vologodskii, A. V. (2011) Temperature dependence of DNA persistence length. *Nucleic Acids Res.,* **39**(4), 1419–1426.

57. Du, Q., Smith, C., Shiffeldrim, N., Vologodskaia, M. Y., and Vologodskii, A. V. (2005) Cyclization of short DNA fragments and bending fluctuations of the double helix. *Proc. Natl. Acad. Sci. U. S. A.,* **102**(15), 5397–5402.

58. Vlahovicek, K. (2003) DNA analysis servers: plot.it, bend.it, model.it and IS. *Nucleic Acids Res.,* **31**(13), 3686–3687.
